# Effective Model Reduction Scheme for the Electronic Structure of Highly Doped Semiconducting Polymers

**DOI:** 10.1021/acs.jctc.4c01131

**Published:** 2024-11-04

**Authors:** Suryoday Prodhan, Alessandro Troisi

**Affiliations:** 1 Department of Chemistry, University of Liverpool, Liverpool L69 3BX, U.K.; 2 Department of Chemistry, Birla Institute of Technology and Science, Pilani, Hyderabad Campus, Hyderabad 500078, India

## Abstract

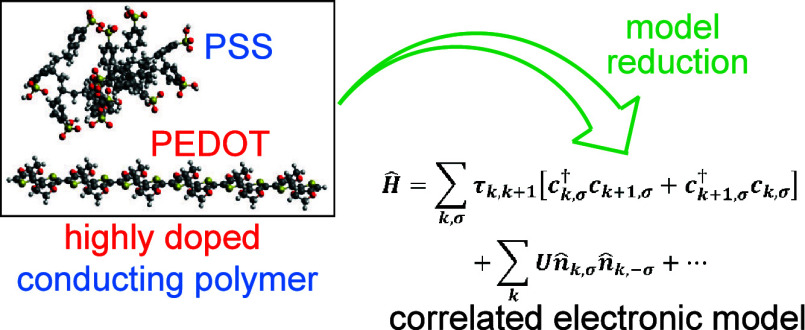

Highly doped organic polymers have emerged as prominent candidates within novel technological disciplines, yet the fundamental correlation between structure and charge transport characteristics still remains missing. Toward this objective, an efficient model reduction scheme for highly doped polymer chains is developed considering the paradigmatic case of poly(3,4-ethylenedioxythiophene)-poly(styrenesulfonate) (PEDOT–PSS). The reduced model accounts for the chemical and structural details of the conducting polymer chain in addition to the long-range Coulombic interactions between charge carriers (holes) and dopant ions and the Coulombic repulsion between holes residing on the PEDOT chain. The model is shown to reproduce the intrachain hole-density profile of bulk polymer chains within a mean-field description. Furthermore, and critically, the model is adept at determining the energy distribution of doped PEDOT samples that in effect, influences the hole distribution among polymer chains. The hole distribution so obtained broadly upholds the approximation of a homogeneous charge-carrier distribution in doped polymers commonly found in the literature. In addition, it is observed that the spin configuration of the charge carriers dictates the energetics of the doped chains while it is a critical function of the chain length, carrier density, and disorder parameters.

## 1 Introduction

The advent of the electrochemical doping of conjugated organic polymers opens new horizons in organic semiconductor technologies.^1,2^ The electrochemical pathway allows the injection of ions from an electrolyte into the organic film and thus allows bulk doping of the organic semiconductor compared to the field-effect doping in conventional organic field-effect transistors. The coupling between the ions and charge carriers within the entire volume of the polymer modulates the bulk conductivity and enhances the transconductance of the devices. In recent years, novel ion-exchange doping techniques have also been developed which achieves very high ion-exchange efficiency and can result in carrier density close to the density of molecular repeating units in conducting polymers.^3^ However, high carrier density usually modulates the intrinsic transport mechanism within organic semiconducting materials as demonstrated by the reduction in activation energy;^4−7^ enhanced dopant concentration boosts thermally activated hopping of the charge carriers as well as the release of free carriers through collective screening.^7^ Early experiments on electrochemically doped conjugated polymers have shown an improvement in conductivity with increasing carrier density at comparatively low carrier concentrations, while a maximum in conductivity is observed at higher doping limits.^8−10^ This observation is independent of the electrolytes used^10^ and it has been reported that the decline in conductivity does not result from the structural deterioration of polymers. Hall resistance measurements have established that charge transport within these conducting polymers is decoherent in nature as opposed to the state-of-the-art polymer poly(2,5-bis(3-tetradecylthiophen-2-yl)thieno[3,2-*b*]thiophene) (PBTTT), though conductivity values are of the same order of magnitude in both systems.^11^ Recent findings about the role of various other factors, *e.g.,* Coulombic traps due to dopant ions,^12−14^ paracrystallinity of the conducting grains,^14^ processing-induced morphology changes,^15,16^ side-chain engineering,^17,18^ molecular weight,^19^ and doping gradient,^20^ on charge-carrier transport have brought about a great deal of attention in the scientific community, yet, charge transport mechanism within highly doped polymers still remains as a fundamental question to be investigated in detail.

Early theoretical description of charge transport in highly doped polymers has frequently been modeled within Efros–Shklovskii variable-range hopping model^21,22^ or been based on the formation of bipolaron bands at high dopant density.^9^ In the mid-2000s, Arkhipov et al. proposed a phenomenological model considering a Gaussian density of states to explain the experimental findings;^23−25^ however, these proposed models employ empirical parameters extracted from fitting experimental results^11,26^ and do not correlate transport characteristics with the chemical structures of the polymers. The Arkhipov model also neglects the mutual Coulombic repulsion between charge carriers and has been found to be effective only up to moderate dopant concentrations (∼10% doping level). Recent kinetic Monte Carlo (KMC) simulations of prototypical semiconductor molecules have demonstrated the doping-induced modifications in the Fermi level positions,^27^ charge conductivity,^27,28^ carrier mobility,^29,30^ and Seebeck coefficient.^31,32^ These studies include a full treatment of the many-body Coulombic interactions between carriers and dopants and illustrate that the consequence of carrier–carrier Coulombic interactions is larger compared to dopant–carrier interactions on charge-carrier transport.^28^ Despite this, these qualitative treatments do not specify how chemical structures affect carrier localization and electronic conductivity in different polymers.

First-principles studies of highly doped organic polymers are usually limited by the higher computational cost and restricted to moderate-sized systems, i.e., oligomers.^33−36^ In the recent past, a number of studies have been reported that calculate electronic conductivity in doped polymers within band theory (uncorrelated charge-carriers) employing the Boltzmann transport equation.^34,37−39^ Comin et al. computed the energy levels of 2,3,5,6-tetrafluoro-7,7,8,8-tetracyanoquinodimethane (F4TCNQ) doped PBTTT within hybrid quantum/classical (QM/MM) calculations to assess the role of dopant position within the polymer lamellae on free charge-carrier generation, where only three PBTTT monomer units along with the F4TCNQ molecule are treated quantum mechanically.^40^ To deduce the structure-transport correlation, Muñoz et al. have modeled the electronic structures of PEDOT oligomers considering the realistic morphology of the chains and a semiempirical Hamiltonian with atomic parameters, yet the atomic basis of the Hamiltonian constrains the size of the system that can be probed.^41^ It is only very recently that theoretical studies of highly doped polymers have extracted transport parameters from first-principles calculations, and calculated charge conductivity considering both dopant-carrier and carrier–carrier interactions; yet the model either adopts spinless nature of the charge-carriers in their description, hence neglects double charge-carrier occupation in the monomer units,^14^ or represents the polymer system in a spin-restricted formalism and ignores the electronic correlation between charge-carriers of different spins.^42^

Understanding the charge-transport mechanism in highly doped polymers is challenging since the carrier-transport pathways are the sum results of intrachain, interchain, and interdomain transport. Even at the intrachain level, the combined effect of conformational disorder, electrostatic disorder, electronic correlation, and ion dynamics in the polymer-ion system influence the charge-carrier dynamics. To make progress in realizing the charge transport mechanism, it would be ideal to develop a reduced model that can capture disorder (spatial as well as energetic) and correlation effects in the polymer system while remaining tractable. The importance of these bottom-up approaches is evident from the analysis by Kang and Snyder,^43^ who observed a fundamental difference in charge transport mechanisms between PEDOT-based polymers and conventional, doped polymers (P3HT, PBTTT) despite having structural similarity. Investigations of the underlying transport mechanism also ask for fast computation of the energetics of the charge transfer process between chains, which is not feasible presently within first-principles techniques. On the other hand, the well-known disorder models^24,44^ of semiconducting polymers employ a single, averaged parameter to characterize disorder within these systems which can be of significant magnitude,^11,26^ of various origins,^45^ and not necessarily uncorrelated.^14^ In regard to the electronic correlation effects, KMC methods do not take into account the interplay between carrier delocalization and on-site carrier–carrier correlation,^27,28^ while analytical solutions of electronically correlated models^46−52^ (Hubbard or extended Hubbard) are primarily limited to specific doping concentrations (quarter-filled or half-filled) with equivalent sites, which do not represent realistic polymers where the doping and consequently the number of charge-carriers can be varied in a more continuous manner. Thus, in the expanding field of highly doped polymers, a robust bottom-up computational scheme is crucial to correlate the chemical structure of the polymers with their transport characteristics.^53,54^

Toward this missing link, we have developed an efficient computational scheme that can model highly doped polymers with varying disorders and carrier densities (Section 2). We consider the state-of-the-art PEDOT–PSS polymer blend as a model system where electronic transport occurs along the PEDOT chains. The effective model reduction scheme can correlate the chemical structures of these highly doped polymers with their electronic characteristics like carrier localization length and electronic conductivity in a computationally inexpensive way while maintaining the many-body nature of the essential parameters all along. The reduced model captures the effects of torsional disorder, electrostatic disorder, and hole–hole correlation while maintaining the criterion that a mean-field description within the effective model should represent the intrachain charge-density profile of the polymers in the bulk, i.e., in the presence of other polymer chains and multiple counterions. To demonstrate the potential of the model developed, we determine the distribution of energies of a sample of variably doped PEDOT-12 chains, that can, in fact, establish the charge distribution among the polymer chains in a realistic sample. “Equal doping per chain” approximation, i.e., the assumption of an equal number of charge carriers on each polymer chain is generally considered in the literature^55−59^ in view of the high computational cost of calculating the energies of individual polymer chains in a disordered sample at variable charged state within ab initio formalism; however, the similar order of magnitude of the transfer integral, on-site energy disorder and repulsive Coulomb potential are expected to determine the oxidation states of the polymer chains in a more subtle way. In addition, the spin degree of freedom becomes important in highly doped polymers, and to that effect, we first assess the importance of spin configuration in extracting the correct energetics of the heavily doped PEDOT chains (Section 3). In Section 4, we employ those outcomes along with the reduced model to compute the ground state charge distribution in highly doped bulk PEDOT samples and examine the validity of the equal doping approximation.

## 2 Model Reduction Scheme and Effective Hamiltonian

We resort to a HOMO-only description of the electronic structure of the highly doped polymers, as was successfully adapted for low-carrier density donor–acceptor copolymers,^60^ assuming that the valence band of the polymer can be well-represented by a linear combination of HOMO orbitals on individual monomer.^61^ We consider the polymer chains as well as the counterions in frozen configuration while distortion of the polymer chains from ideal planar conformation is also taken into account; subsequently, the effective model is representative of the bulk system at absolute zero temperature. The charge neutrality of the bulk polymer system also sets that the total number of counterions is equal to the total number of holes in the system.^14^

The reduced Hamiltonian for highly doped polymer chain is given by,
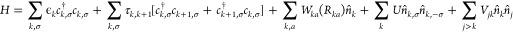
1where *c*_*k*, σ_^†^ (*c*_*k*, σ_) creates (annihilates) a hole with spin σ at the *k*-th monomer HOMO, the spin index (σ) drops only if a single hole is present on the polymer chain. *n̂*_*k*, σ_ = *c*_*k*, σ_^†^*c*_*k*, σ_ is the occupation number operator of σ-spin at *k-*th monomer HOMO while *n̂*_*k*_ = *n̂*_*k*, σ_ + *n̂*_*k*,−σ_ is the total occupation number operator of the *k*-th monomer HOMO with highest expectation value of 2 (otherwise it violates Pauli’s exclusion principle for Fermions), i.e., each monomer HOMO basis can accommodate a maximum of 2 holes with opposite spins. ϵ_*k*_ is the HOMO energy (on-site energy) of the *k-*th monomer in the chain in the absence of any counterion; for the homopolymer PEDOT, we consider ϵ_*k*_ = 0. τ_*k*, *k*+1_ is the electronic coupling between monomer *k* and *k* + 1, which observes a sinusoidal relationship with the corresponding dihedral angle θ_*k*, *k*+1_ (see SI Figure S1)
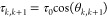
2

*W*_*ka*_ is the electronic potential energy of a hole located at the center of charge of *k*-th monomer HOMO due to the counterion *a* at a distance *R*_*ka*_ (the center of charge of 3,4-ethylenedioxythiophene (EDOT)/styrenesulfonate (SS^–^) is the weighted geometric center of the corresponding moiety, the weights being contributions of each atom to the corresponding HOMO orbital as determined by Multiwfn analyzer^62^); in essence, *W*_*ka*_ represents the modulation in the on-site energy of monomer *k* due to the presence of the counterion *a*. *U* is the Hubbard-type energy term which contributes to the Hamiltonian if the *k*-th HOMO orbital is doubly occupied, while *V*_*jk*_ is the long-range Coulombic interaction term between holes located at monomers *j* and *k*. For simplicity, we considered Ohno parametrization scheme^63−66^ for *V*_*jk*_
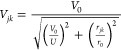
3a
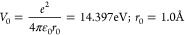
3bwhich interpolates between *U* at *r*_*jk*_ → 0 and *e*^2^/4πε_0_*r*_*jk*_ = *V*_0_/(*r*_*jk*_/*r*_0_) for *r*_*jk*_ → ∞. In eq 3a and 3b, *V*_*jk*_ and *U* are expressed in eV units while *r*_*jk*_ is the distance between the center of charges of monomers *j* and *k*; *e* and ε_0_ are the electronic charge and the vacuum permittivity, respectively.

From the above discussion, it is evident that the effective model of highly doped polymer is represented by the two parameters τ_0_ and *U* only (eqs 1–3b), and in the following subsections, we detail the computational workflows for determining these parameters.

In the present model, we have not considered the electron–phonon coupling since it is well-accepted in the organic semiconducting polymer community that the localization of the charge-carriers does not stem from the polaronic effects but rather from the static disorder in the intermonomer electronic coupling by virtue of the soft nature of the polymer backbone,^67,68^ and the charge-carrier transport can be considered as a series of hopping events between the localized states.^69−71^ Since the static disorder in the electronic coupling is larger in magnitude compared to the monomer’s reorganization energy, the effect of the electron–phonon coupling can be introduced as the first-order correction after determining the eigenstates of the proposed model Hamiltonian (eq 1);^60^ the delocalization length of the eigenstates rescales the monomer reorganization energy. Experimental measurements of the charge-carrier mobility in different polymers also validate that it is correlated with the structural disorder but not with the electron–phonon coupling strength.^67^ Since the coupling between the electronic degrees of freedom and the nuclear vibration, in general, regulates the rate of charge hopping between the localized states,^68,72^ the exclusion of this effect does not affect the inference drawn from the present study on the localization of charge carriers. However, electron–phonon coupling will be essential in the continuation of the present work for the computation of hopping rates and to probe the charge-carrier dynamics.

### 2.1 Fitting of Single-Hole Parameters

**Figure 1:**
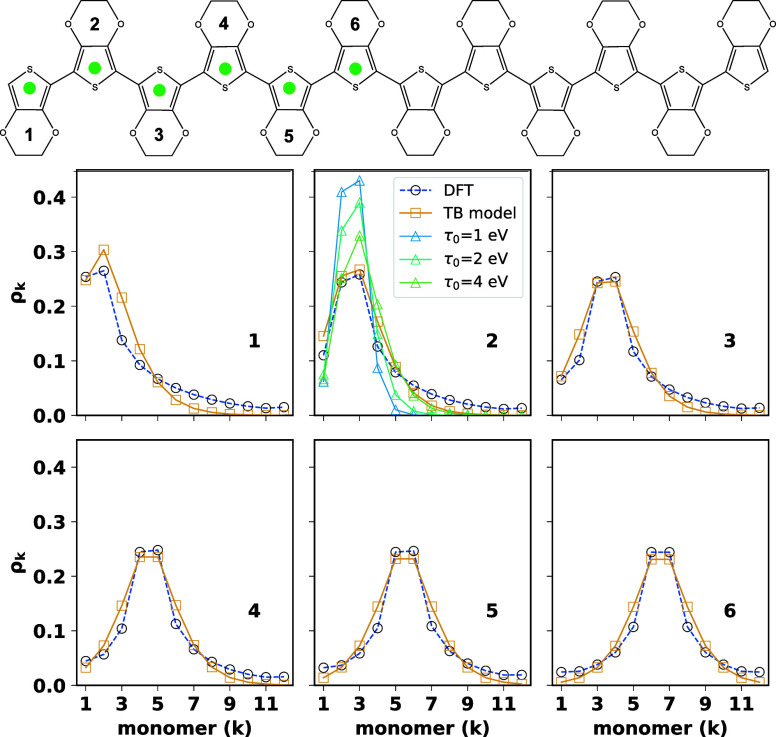
Monomer hole denisty profile in PEDOT-12 chains calculated within DFT (blue broken, circle) and fitted within the TB model (orange solid, squre). The single negative counterion is placed 4.0 A° above the molecular plane. Different ion positions (green solid spheres) with respect to PEDOT monomers are shown schematically in the top subfigure. The middle and bottom panels render the qualitative agreement between ρ_*k*, DFT_ and ρ_*k*, TB_ on employing the optimal single-hole parameters. For ion position 2, monomer hole desnity profiles for τ_0_ = 1.0, 2.0, and 4.0 eV, without introducing ϵ_chain end_, are also plotted (second subfigure of the middle panel) which shows significant difference in the fitting; the corresponding color and symbol indices are given in the subfigure inset. In the last three scenarios, ϵ_chain end_ is not introduced to avoid artifacts.

We start the model reduction by first determining the effective τ_0_ between PEDOT monomer HOMOs—the single parameter, which regulates intrachain hole transport in singly doped PEDOT chains within the present model. The efficacy of τ_0_ to describe singly charged PEDOT chains, in the presence of a single counterion (considered as a point charge in the background), is evident from the qualitative reproduction (see Figure 1) of the intrachain monomer hole density profiles (ρ_*k*_) obtained at the density functional theory (DFT) level (ρ_*k*, DFT_) within a tight-binding (TB) model Hamiltonian (ρ_*k*, *TB*_) consisting of the first two terms in eq 1. We consider PEDOT chain with 12 monomer units (PEDOT-12), geometry-optimized at the DFT level, and strategically place the counterions above the molecular plane at a distance of 4 A°; both geometry optimization as well as the single point calculations at the optimized geometry of PEDOT-12 in the presence of the negative ions are done at the B3LYP/3-21G* level using Gaussian-16 package suit.^73^ As optimized PEDOT-12 exhibits an almost planar structure with the monomer units in anticonformations, ρ_*k*_’s dependence on θ_*k*, *k*+1_ becomes irrelevant and τ_*k*, *k*+1_ = −τ_0_ is a constant here.

Optimal value of τ_0_ (τ_0_^opt^) is computed by minimizing the root mean squared error (RMSE, eq 4) in monomer hole density, averaged over 12 different counterion conformations (⟨RMSE⟩_ion_).
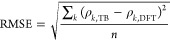
4

*n* is the number of monomers in the corresponding PEDOT chain. Within the DFT formalism, ρ_*k*, DFT_ is calculated by ρ_*k*, DFT_ = ρ_*k*, Mulliken_^charged^ – ρ_*k*, Mulliken_^neutral^, ρ_*k*, Mulliken_^charged(neutral)^ being the total Mulliken charge on monomer *k* in the singly charged (neutral) PEDOT chain. On the other hand, ρ_*k*, TB_ is calculated from the TB ground state ψ_0_ = ∑_*k*_ψ_*k*,0_ | *k*⟩ (ρ_*k*, TB_ = |ψ_*k*,0_|^2^) obtained by diagonalizing the TB Hamiltonian matrix with open boundary conditions (OBC). Due to the OBC, ψ_*k*,0_ is usually smaller toward the chain ends within the TB formalism,^74^ and subsequently, for further improving ρ_*k*, TB_, we introduce an on-site energy correction for the chain end monomers (*i.e.,* ϵ_1_ = ϵ_*n*_ = ϵ_chain end_) (see SI Figure S2).

We simultaneously determine τ_0_^opt^ and ϵ_chain end_^opt^ considering 12 counterion configurations, each at 4 *A*° distance from the molecular plane, employing a dual-annealing algorithm within SciPy package.^75^ The computational workflow for optimizing the single-hole parameters is shown in SI Figure S4, while the range of parameter values of τ_0_ and ϵ_chain end_, set for the dual annealing optimization, are given in SI Table S1. We find that at optimal τ_0_ ≈ 6.1 eV and ϵ_chain end_ ≈ 3.9 eV, ⟨RMSE⟩_ion_ = 0.025 i.e., the average error in monomer hole density fitting is only ∼2.5%.

The τ_0_ value obtained through the above fitting procedure is larger compared to the inter-*p*_*z*_ transfer integral for conjugated molecules (∼2.4 eV)^76,77^ or the HOMO–HOMO coupling extracted from the bandwidth within DFT calculations.^34,78^ Given the very low-dimensional model Hamiltonian, the parameter τ_0_ in the present scenario does not represent the HOMO–HOMO coupling between PEDOT units but renormalizes all electronic interactions into one effective parameter. To verify that this is not an artificial effect (could in principle be caused by overfitting) we show in an additional test that using smaller τ_0_ values does not reproduce the charge density of an excess hole; as an example, for counterion position 2 (see top panel of Figure 1), *RMSE* in the monomer hole density (eq 4), without introducing ϵ_chain end_ (to avoid fitting artifacts), is 7.7, 5.4, 3.6, and 3.8% for τ_0_ = 1.0 eV, 2.0, 4.0, and 6.1 eV, respectively (see Figure 1, middle panel, second subfigure). Furthermore, as we are primarily interested in accurately predicting the localization of the excess charge, τ_0_ is derived by fitting the monomer hole density rather than the valence bandwidth.

### 2.2 Efficiency of Reproducing in Bulk Semiconductor

Since parametrization of the single-hole parameters has been carried out considering a fixed separation of the counterions from the polymer chain, we further check the accuracy of the scheme by considering 40 random SS^–^ ion placements around the PEDOT-12 chain. To model realistic polymer-ion systems, the typical distance of SS^–^ ions from PEDOT backbones is extracted from the atomistic MD simulation outputs of PEDOT–PSS blends^58^ and employed to generate the random ion coordinates. We observe ⟨RMSE⟩_ion_ = 0.025 on considering 40 random SS^–^ ion configurations with PEDOT-12, that is, similar to the earlier computed ⟨RMSE⟩_ion_ with fixed separation of the counterions. Repeating the calculations with PEDOT chains of varying length (PEDOT-6 and PEDOT-18), we observe that the ⟨RMSE⟩_ion_ values are 0.044 and 0.021, respectively; the larger error in fitting for smaller PEDOT chain is expected from finite-size effects.^74^

These calculations with random, realistic SS^–^ ion configurations further justify the model Hamiltonian (eq 1) and the single-hole parameters which reproduce monomer hole density profile within ∼5% error for realistic bulk polymer-ion systems (see SI Figure S3). Indeed, there is no fundamental limitation over the use of a larger set of parameters for more quantitative fitting of ρ_*k*, DFT_ profiles, yet, in this work we focus on the development of a reduced model, which qualitatively reproduces ρ_*k*, DFT_ profile and offers much better control. The minor adjustments of the methodology can be implemented on a case-by-case basis, while a more general description will be convenient for high-throughput screening studies considering the large set of candidate polymers.

### 2.3 Fitting of Hole–Hole Coulombic Interaction Parameter

We employ the single-hole parameters so obtained to estimate *U* by fitting *ρ*_*k*_ in PEDOT-12 chains with multiple charge-carriers, calculated at the DFT level (B3LYP/3-21G*), within the model Hamiltonian (eq 1) at the mean-field level (in both calculations, optimized geometry of neutral PEDOT-12 chain is considered). We employ a similar dual annealing workflow, as for single-hole parameters, to optimize *U* by minimizing ⟨⟨RMSE⟩_ion_⟩*_N_h__*, where *RMSE* is averaged over varying numbers of holes in the chain (*N*_*h*_ = 2, 3, and 4) and 25 multiple counterion configurations for each *N*_*h*_ (number of counterions in each configuration is same as *N*_*h*_). The range of *U* set at the onset of the dual annealing is given in SI Table S1.

To compute the one-particle wave functions of the interacting model at the mean-field level, we adopt the available Fortran program developed by Sony and Shukla for probing electronic structures of π-conjugated molecules within correlated electronic models.^79^ Since the current model include the spin degrees of freedom of the charge carriers, we resort to the unrestricted solution of the Hamiltonian, i.e.*,* μth eigenstate for α and β spins (ψ_μ_^α^ and ψ_μ_^β^ respectively) are represented by linear combinations of EDOT HOMO orbitals ({ϕ_*k*_}) with differing coefficients (). We assume that {ϕ_*k*_} corresponds to an orthonormal basis set and the overlap between neighboring monomer HOMO orbitals is small (*S*_*kj*_ ≈ δ_*kj*_). The spin orbitals of the interacting Hamiltonian are determined by iteratively solving the Pople-Nesbet equations for the corresponding spin^80^ (strategies to achieve convergence within the SCF formalism are detailed in the SI),
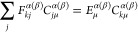
5where *E*_μ_^α(β)^ is the μth α(β)-spin–orbital energy and *F*_*ij*_^α(β)^ is the corresponding Fock matrix. The matrix elements of the Fock matrices *F*^α^ and *F*^β^ are given by (full derivation is given in the SI),
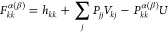
6
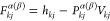
7with *h* being the single-hole Hamiltonian. *P*^α^, *P*^β^, and *P* = *P*^α^ + *P*^β^ are the α- and β-spin density matrices and the total density matrix, respectively.
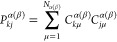
8

In eq 8, *C*_*k*μ_^α(β)^ values are considered real while *N*_α_(*N*_β_) is the number of occupied α(β)-spin orbitals which maintains the relation *N*_α_ + *N*_β_ = *N*_*h*_. In eq 6, the second term on the RHS designates the Coulomb term of the Fock operator, which contains interactions between holes of both α- and β-spins, while the last term (exchange term of Fock operator) can only have contributions from orbitals with the same spin.

The optimal values of the parameters, obtained via fitting *ρ*_*k*_ in PEDOT-12 with 2, 3, and 4 holes per chain within the model Hamiltonian (eq 1) (⟨⟨RMSE⟩_ion_⟩*_N_h__* ≈ 0.04), are tabulated in Table 1. The computational workflow for optimizing the multiple-hole parameter is shown in SI Figure S5, while the representative fittings of ρ_*k*_ in multiple-hole PEDOT chains are presented in SI Figure S6.

**Table 1:** Optimal Values of the Parameters of the Effective Model Hamiltonian for the PEDOT Polymer

**parameter**	**optimal value (eV)**
τ_0_	6.146
ϵ_chain end_	3.942
*U*	8.202

**Figure 2:**
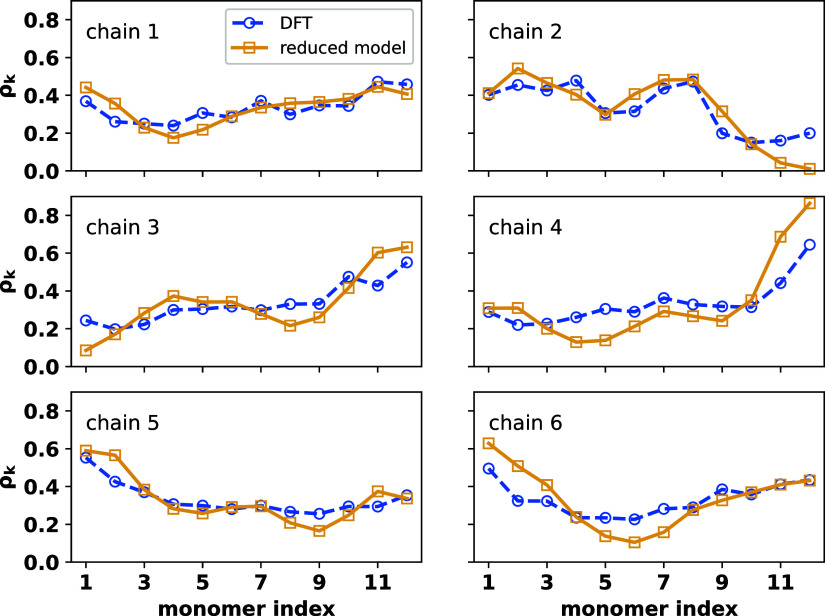
Monomer hole density profile calculated within DFT (blue broken, circle) and reproduced within the reduced model Hamiltonian (eq 1) (orange solid, square) for PEDOT-12 chains with 4 holes on each chain and in the presence of 480 SS^–^ ions and 119 PEDOT-12 chains with +4 charge. Each monomer of the 119 PEDOT-12 chains in the background is assumed to correspond to a positive point charge of charge density +0.33*e*.

The efficiency of the developed parametrization scheme is tested by reproducing ρ_*k*, DFT_ for six PEDOT-12 chains with 4 holes on each, extracted from an earlier work^58^ with 120 PEDOT-12 chains and 480 SS^–^ ions present in the simulation box, employing the effective model Hamiltonian. DFT calculations over the PEDOT-12 chain with multiple holes are done in their unoptimized conformations with all negatively charged counterions present as background charges. The respective PEDOT-12 chains are assumed to have unoptimized geometries to represent realistic chains in bulk semiconductors. We also consider the remaining PEDOT chains in the simulation box (excluding the particular PEDOT-12 chain in question) as background with a positive charge density of ∼0.33 on each monomer unit. Mulliken charges over each monomer in the corresponding neutral PEDOT-12 chain are calculated by excluding four randomly chosen SS^–^ ions closest to the PEDOT-12 chain from the simulation box. The hole density profiles calculated employing the model Hamiltonian (Figure 2) show qualitative agreement with ρ_*k*, DFT_ with an average RMSE over six PEDOT-12 chain conformations ⟨RMSE⟩_conform_ ≈ 0.08. It should be noted that the DFT calculations are done on an unoptimized chain geometry which may induce error in ρ_*k*, DFT_, yet the qualitative agreement between the two results points toward an effective model reduction of these highly doped systems.

## 3 Spin Configuration in Doped and Disordered PEDOT Chains

The charge-carrier distribution as well as the charge-carrier transport in realistic polymer systems depends on the energy landscape of the localized states. However, the spin degree of freedom of the charge carriers becomes important in the many-body description that modulates the energy of the localized states, and the energy value does not follow a trivial relationship with the spin configuration of the charge carriers. Consequently, ignoring the spin description leads to erroneous results on the distribution of charges in the bulk polymer system (at least in some relevant cases). Hence, with the reduced model developed above and with the knowledge of electrostatic and conformational disorders of PEDOT chains in a realistic system, we first explore the ground state spin configuration of doped PEDOT chains and examine how the spin state influences the total energy of the highly doped chains.

We compute SCF energies of PEDOT chains with *n* monomers (*n* = 6, 12, 18, and 24) at varying doping levels (up to one hole per monomer, a single hole in the chain is not considered) and determine the ground state spin configuration at a particular doping level *N*_*h*_ by varying *N*_α_ and *N*_β_ while strictly maintaining the condition *N*_α_ + *N*_β_ = *N*_*h*_ (see Section 2 for the definition of *N*_*h*_, *N*_α_, and *N*_β_).^81−84^ We emphasize that in the present scenario, ground state spin configurations within the chains arise due to electronic correlation in the reduced Hamiltonian (eq 1), not from spin–spin interaction between carriers located on different chains. We also acknowledge that it is not possible to accurately determine the total spin (*S*) of individual polymer chains with a particular spin configuration (*M*_*S*_ = 0.5 × (*N*_α_ – *N*_β_)) within the unrestricted mean-field description.^85^ However, it has been shown within the correlated electronic model studies^86,87^ that the vanishing energy gap between the lowest energy configurations with *M*_*S*_ ≥ 0 and *M*′_*S*_ = *M*_*S*_ + 1 implies a ground state with total spin *S* = *M*′_*S*_. For each *N*_*h*_, we consider the cases of *N*_α_ ≥ *N*_β_ only, since we find that the difference in SCF energies for *M*_*S*_ = +*M* and *M*_*S*_ = −*M* is negligible and they represent the same *S* state (in the absence of an external magnetic field).

To introduce torsional disorder in the simulations, the torsional potential of bulk PEDOT chains is determined (shown in SI Figure S7) and further parametrized (outlined in ref (60)), and the dihedral angle between neighboring PEDOT monomers is randomly drawn from the corresponding Boltzmann distribution. To simplify the description of the polymer system, we also ignore individual counterions and randomly assign the PEDOT monomer HOMO energies from a Gaussian distribution, centered at zero, with a width of ∼1.5 eV (justification is given in SI Figure S8). The effect of disorder is verified by considering an ensemble of 100 PEDOT chains for a particular {*N*_*h*_, *N*_α_, *N*_β_}, and we report the distribution of the ground state spin configurations of the PEDOT chains.

**Figure 3:**
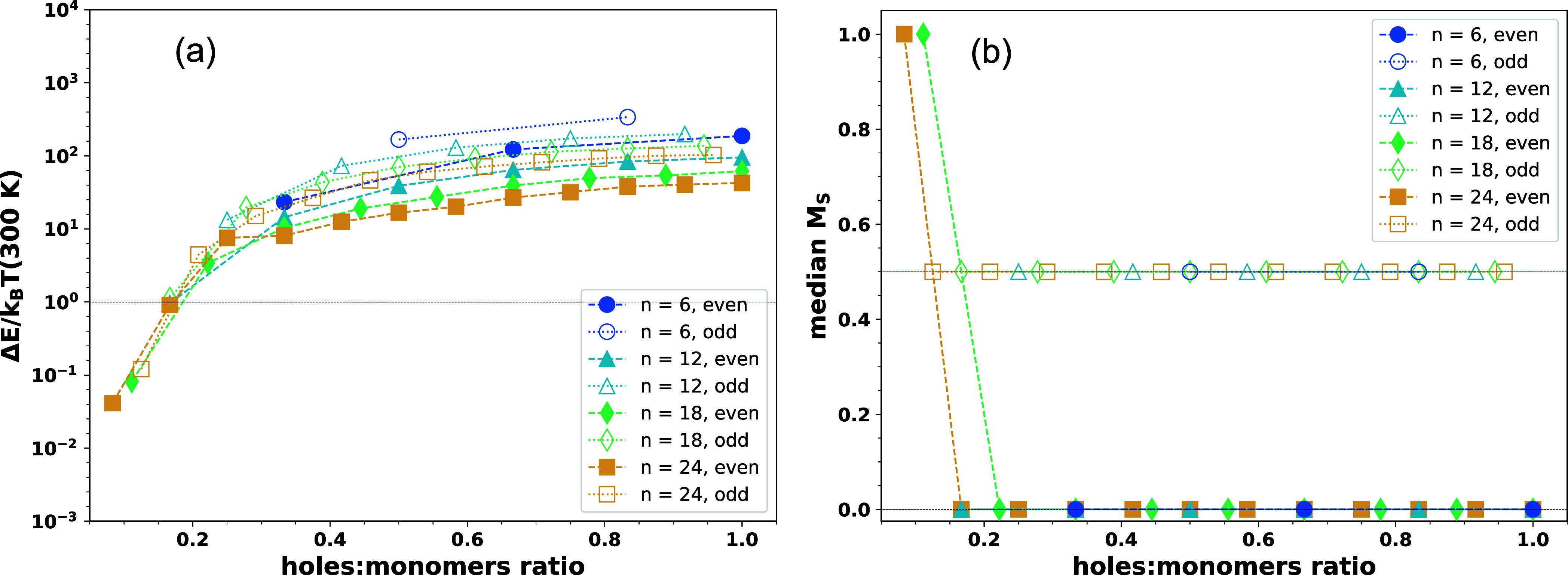
(a) Ensemble-averaged energy gap between ground state spin configuration and next higher energy spin configuration in PEDOT chains of varying length and with varying numbers of holes. The color and symbol indices are given in the figure inset where the filled (open) symbols represent even (odd) number of holes in the chain. The energy gaps are represented in units of *k*_*B*_*T*, *T* = 300 K, and plotted in the logarithmic scale. The black dotted line represents Δ*E* = *k*_*B*_*T*; other lines connecting the symbols are guides to the eyes. (b) Median of the ground state *M*_*S*_ distribution of PEDOT chain ensembles of specific chain length and with a particular number of holes. Color and symbol indices are similar to panel (a), and lines connecting the symbols are guides to the eyes. The black (red) dotted line represents *M*_*S*_ = 0 (0.5), i.e., a singlet (doublet) configuration.

In Figure 3a, we present the ensemble-averaged energy gap between the ground state spin configuration and the next higher energy spin configuration for different doping levels and varying PEDOT chain lengths, while in Figure 3b, we show the corresponding median value of the ground state *M*_*S*_ distributions of the PEDOT chain ensembles (distributions of the ground state *M*_*S*_ values of the ensembles are shown in SI Figure S9). The energy gaps between the low-energy spin configurations (Figure 3a) are a measure of the propensity of thermal fluctuations among the spin configurations while the ground state *M*_*S*_ distributions (Figure 3b and SI Figure S9) indicate how resilient the ground state spin configuration is toward the torsional disorder of PEDOT chains as well as the electrostatic disorder due to random counterions. As it is evident from the latter, for hole densities of ≳0.5, the system primarily assumes the low-spin configuration, i.e., singlet (doublet) configuration for an even (odd) number of carriers. It is also noted that for *N*_*h*_ = *n* cases, the ground state *M*_*S*_ values do follow the well-known Lieb’s theorem^88^ (*viz. S* should be strictly zero), despite the presence of counterions in polymer surroundings that break the particle–hole symmetry. On the other hand, for lower hole densities (≲0.5) the polymer chain can attain high-spin configurations, i.e.*, M*_*S*_ > 0 (0.5) for an even (odd) number of holes. The propensity for high-spin configurations increases with longer PEDOT chains (see SI Figure S9) since it is easier for an α(β)-spin-charge carrier to avoid the Fermi holes of other charge carriers with the same spin over the polymer chain; similar observations have been reported in the unrestricted spin DFT calculations of PEDOT-12 and PEDOT-18 with up to +6*e* charge.^35^ The energy gaps are larger with an odd number of carriers, compared to the even ones, since flipping of carrier spins from α to β (or vice versa) leads to a greater mismatch between *N*_α_ and *N*_β_ in the former cases, which in effect further destabilizes the system (presence of Fermi holes^85^). It should also be mentioned that structural and energetic disorders play significant roles in determining the energy gaps, which is evident from the spread of energy gaps obtained for the ensemble of PEDOT chains (see SI Figure S10).

A closer look into Figure 3 reveals that the ground state spin configuration, and in effect, the ground state energy of the charged polymer is a critical function of the chain length, hole density, as well as disorder parameters, and all the components should be included in the polymer description for accurately describing charge-carrier transport in highly doped systems. This inference highlights the usefulness of the reduced model developed here. In addition, as the energy gaps between different spin configurations become much smaller (compared to *k*_*B*_*T*) in longer polymer chains at low-carrier densities (Figure 3a), several spin configurations need to be rigorously computed to identify the lowest energy spin configuration while determining the energy distribution in the bulk polymer system.

## 4 Hole Distribution in Bulk PEDOT Chain Samples

Utilizing the reduced description of the PEDOT chains and the inference from the previous section, the distribution of charge carriers among PEDOT chains in the bulk at a fixed level of doping is probed. We assume that the polymer ensemble energetically relaxes via interchain transfer of holes from the *i*th chain to the *j*th chain until the ensemble attains thermal equilibrium. The distribution of energy levels obtained is also relevant for the hole dynamics, and the model reduction scheme can significantly expedite the computational process of determining the full energy landscape of the bulk system while incorporating the structural, chemical, as well as energetic details. We note that the following strategy is pertinent in determining the lowest-energy distribution of holes, not to reproduce the hole dynamics since we assume that the relaxation of the polymer ensemble is fully dictated by the energetics of the molecules and that hopping of holes is allowed between chains at arbitrary distances.

For the present simulations, we consider fixed electrostatic and conformational disorders of the PEDOT chains and ignore hole–hole interaction across multiple chains, although it is not exceedingly difficult to consider realistic snapshots of the system where the redistribution of charges modulates the on-site energies. We compute the energy of reduction Δ*E*_*i*_^(*R*)^ = *E*_*i*_^(*p* – 1)+^ – *E*_*i*_^*p*+^ (hole removal) and oxidation Δ*E*_*i*_^(*O*)^ = *E*_*i*_^(*p* + 1)+^ – *E*_*i*_^*p*+^ (hole addition) of each chain *i* where *E*_*i*_^(*p* – 1)+^, *E*_*i*_^*p*+^, and *E*_*i*_^(*p* + 1)+^ represents the ground state energy of the *i*th chain with (*p* – 1), *p*, and (*p* + 1) holes residing on the chain, respectively. In calculating *E*_*i*_^*p*+^ and similar quantities, we assume that the PEDOT chains remain in their lowest energy spin states during the hole transition. The rate of a hole hopping from chain *i* to chain *j* is determined by a Miller–Abrahams type rate equation^29,89^
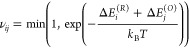
9

In eq 9, we consider both intrinsic hopping frequency as well as the spatial overlap factor between different chains to be unity. Charge-carrier redistribution via hopping is performed within a Monte Carlo approach^89^ following the probability of carrier hopping from chain *i* to chain . Following the redistribution of charges, *E*_*i*_^(*p*^′^ – 1)+^, *E*_*i*_^*p*^′^+^ and *E*_*i*_^(*p*^′^ + 1)+^ for chain *i* are recalculated before proceeding to the next iteration; the above relaxation protocol is repeated until we meet the convergence criteria. To accelerate the relaxation protocol, we allow the hopping of multiple holes between polymer chains at each iteration step, provided that no chain takes part in the allowed hopping processes more than once.

In the simulations, we do not consider a neutral chain within the ensemble i.e., each PEDOT chain at least carries a single charge carrier while we ensure that Pauli’s exclusion principle is also maintained, i.e., the maximum number of charge carriers on a particular chain cannot exceed twice the number of monomer units. We consider an ensemble of 120 PEDOT chains, each with 12 monomers, at an average ∼33% doping level i.e., one hole per three monomers; the corresponding doping level is typical both in theoretical modeling^58,90^ as well as in realistic polymer samples.^91^ The initial PEDOT ensemble is generated by randomly assigning an integer number of holes to each chain while maintaining the total number of holes in the system; for statistical inference, we consider 25 different polymer ensembles with distinct initial distributions of holes, and the final carrier density distribution is computed by averaging over these ensembles. The conformational disorder in individual PEDOT chains within each ensemble as well as the on-site energy disorder due to the charged surroundings are parametrically introduced within the reduced Hamiltonian (similar to that in Section 3).

**Figure 4:**
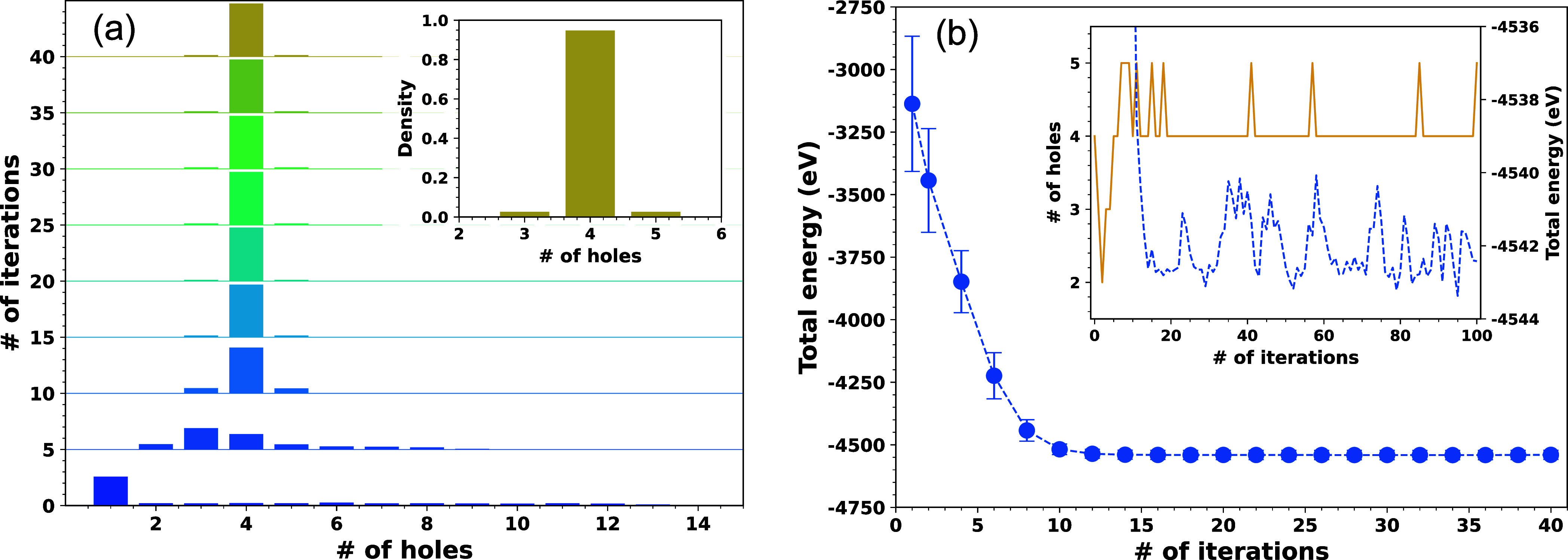
(a) Evolution of the carrier density distribution within PEDOT-12 ensembles on energetic relaxation. The distribution shown is obtained by averaging the carrier density distribution over 25 ensembles with a distinct initial distribution of holes. Inset: magnified view of the distribution after 40th relaxation cycle; it depicts that the distribution does not collapse into a δ-distribution long after the thermal equilibrium is achieved. (b) Development in the total energy of the polymer system suggests that the carrier density distribution saturates after ∼10 relaxation cycles. The mean and standard deviation in total energy of the polymer systems are computed considering 25 ensembles. Inset: evolution of the oxidation state of a randomly chosen PEDOT-12 chain during the relaxation cycle (orange solid curve) and evolution of the total energy of the corresponding PEDOT-12 ensemble (blue broken curve). The fluctuation in the number of holes long after achieving the thermal equilibrium (depicted by the saturation of the total energy of the ensemble) indicates that multiple hole distributions coexist within the energy window of *k*_*B*_*T* and a constant oxidation state of the chain is not maintained.

In Figure 4a, we have shown the evolution of the carrier density distribution on energetic relaxation of the polymer ensembles, averaged over 25 distinctly initiated ensembles. As it can be observed, the distribution gets narrower on energetic relaxation, *yet it does not converge to a system with an equal number of holes over every chain*; in the present case, spread around the maxima of the distribution represents ∼5% of the PEDOT chains (see inset of Figure 4a). The saturation in the total energy (Figure 4b) as well as the perpetually unchanged carrier distribution at the latter part of the relaxation (after ∼10 relaxation cycles; see Figure 4a) signal that the thermal equilibrium has been achieved within each polymer ensemble. Yet, the number of holes on an individual PEDOT-12 chain can fluctuate long after attaining the thermal equilibrium (see inset of Figure 4b). These fluctuations in the hole density of individual PEDOT-12 chains signify that multiple hole distributions coexist within the energy window of *k*_*B*_*T*, and an “equal doping per chain” assumption does not strictly follow. The difference in the hole distribution over individual PEDOT-12 chains results from the combined effects of the electrostatic disorder and the conformational disorder, yet it is difficult to disentangle their individual effect on the hole localization since the synergy between them induces the ruggedness of the energy landscape.

We also draw attention to the fact that in the current simulations, we only consider the energetic bottleneck for the interchain hole transfer, while in realistic polymers it will also be limited by the spatial overlap between different chains. In addition, the deviation from the “equal doping per chain” (3 or 5 holes in the PEDOT chain) is essential for charge transport since there will be no charge-carrier movement without these fluctuations from the average occupation. The “equal doping per chain” assumption is also not applicable at the low doping limit where larger variation in the hole concentration is expected between different chains.

## 5 Conclusions

We have developed an efficient model reduction scheme for highly doped organic polymers that can effectively incorporate chemical as well as structural details of the constituent moieties while considering the long-range Coulombic interactions between holes and counterions as well as the mutual Coulombic interactions between holes on the same chain. In light of the technological importance of these polymeric systems, this work is an endeavor toward including all key physical attributes within an effective model. The reduced model can be explored to investigate electronic features like intrachain charge density profile or charge-carrier localization length, while it will also be effective in discerning the charge-carrier dynamics in these polymers with the aid of a suitable quantum dynamics method. The present work demonstrates that the reduced model can qualitatively (within a ∼10% error limit) predict the electronic structures of multicharged polymer chains in the bulk, i.e., in the presence of counterions as well as other charged polymer chains.

The usefulness of the reduced model is evident from the determination of spin configurations in highly doped PEDOT chains as well as from the extracting of hole distribution in bulk PEDOT-12 samples. Spin multiplicity of doped PEDOT molecules have earlier been probed within DFT formalism, but due to high computational cost it was limited to PEDOT-12 and PEDOT-18 with up to +6 oxidation level;^35^ however, the reduced model developed here does not pose such restrictions on the system size as well as on the number of holes on the chain and indicates that the ground-state spin structure is an intricate function of chain length, number of charge-carriers and the extent of disorder. The reduced model also enables us to verify the “equal charge on every chain” approximation considered in the literature, and we observe that while most of the PEDOT chains retain the same number of holes, the distribution does not shrink to a δ-distribution but yields a finite width long after achieving the thermal equilibrium. The calculation also shows that multiple hole distributions, where the oxidation state of individual polymer chains varies, coexist at thermal equilibrium, and they will play relevant roles in transport physics.

Finally, the reduced description of the polymers also manifests its usefulness in easy transferability to other benchmark polymers and easy-comparison of promising materials; in other words, the model is not limited to the PEDOT–PSS system. Hole dynamics in realistic highly doped polymers can also be probed within the reduced model by including interchain hole–hole repulsion and introducing a prefactor in the Miller–Abrahams rate that reflects the vicinity between chains. The reduced model can also serve as an ideal starting point for multiscale modeling of organic mixed ionic electronic conductors (OMIEC) and organic thermoelectric materials, and the inclusion of the electron–phonon coupling/vibronic effects seems to be the next logical step. These are topics for future research.

## Data Availability

The data that support the findings of this study are available from the corresponding authors upon reasonable request.

## Supporting Information Available

Electronic coupling between EDOT HOMOs (Section S1), fitting of monomer hole density in singly charged PEDOT-12 chains with or without ϵ_chain end_ (Sections S2 and S3), parameter range for dual annealing scheme (Section S4), computational workflow for optimization of single- and multiple-hole parameters (Section S5), fitting of monomer hole density in multiple-hole PEDOT-12 (Section S6), derivation of the Fock matrix elements (Section S7), SCF solutions within UHF calculations (Section S8), optimal damping algorithm for accelerating convergence of SCF solutions (Section S9), torsional potential profile of PEDOT dimer (Section S10), PEDOT monomer HOMO energy distribution (Section S11), distribution of ground state *M*_*S*_ (Section S12), and energy gaps (Section S13) in PEDOT chains of varying length and with varying number of holes (PDF)

## References

[ref1] Rivnay J.; Inal S.; Salleo A.; Owens R. M.; Berggren M.; Malliaras G. G. Organic Electrochemical Transistors. Nat. Rev. Mater. 2018, 3 (2), 17086 10.1038/natrevmats.2017.86.

[ref2] Paulsen B. D.; Tybrandt K.; Stavrinidou E.; Rivnay J. Organic Mixed Ionic–Electronic Conductors. Nat. Mater. 2020, 19 (1), 13–26. 10.1038/s41563-019-0435-z. 31427743

[ref3] Jacobs I. E.; Lin Y.; Huang Y.; Ren X.; Simatos D.; Chen C.; Tjhe D.; Statz M.; Lai L.; Finn P. A.; Neal W. G.; D’Avino G.; Lemaur V.; Fratini S.; Beljonne D.; Strzalka J.; Nielsen C. B.; Barlow S.; Marder S. R.; McCulloch I.; Sirringhaus H. High-Efficiency Ion-Exchange Doping of Conducting Polymers. Adv. Mater. 2022, 34 (22), 2102988 10.1002/adma.202102988. 34418878

[ref4] Shen Y.; Diest K.; Wong M. H.; Hsieh B. R.; Dunlap D. H.; Malliaras G. G. Charge Transport in Doped Organic Semiconductors. Phys. Rev. B 2003, 68 (8), 081204 10.1103/PhysRevB.68.081204.

[ref5] Zhang F.; Kahn A. Investigation of the High Electron Affinity Molecular Dopant F6-TCNNQ for Hole-Transport Materials. Adv. Funct. Mater. 2018, 28 (1), 1703780 10.1002/adfm.201703780.

[ref6] Schwarze M.; Gaul C.; Scholz R.; Bussolotti F.; Hofacker A.; Schellhammer K. S.; Nell B.; Naab B. D.; Bao Z.; Spoltore D.; Vandewal K.; Widmer J.; Kera S.; Ueno N.; Ortmann F.; Leo K. Molecular Parameters Responsible for Thermally Activated Transport in Doped Organic Semiconductors. Nat. Mater. 2019, 18 (3), 242–248. 10.1038/s41563-018-0277-0. 30692647

[ref7] Comin M.; Fratini S.; Blase X.; D’Avino G. Doping-Induced Dielectric Catastrophe Prompts Free-Carrier Release in Organic Semiconductors. Adv. Mater. 2022, 34 (2), 2105376 10.1002/adma.202105376. 34647372

[ref8] Ofer D.; Crooks R. M.; Wrighton M. S. Potential Dependence of the Conductivity of Highly Oxidized Polythiophenes, Polypyrroles, and Polyaniline: Finite Windows of High Conductivity. J. Am. Chem. Soc. 1990, 112 (22), 7869–7879. 10.1021/ja00178a004.

[ref9] Harima Y.; Eguchi T.; Yamashita K. Enhancement of Carrier Mobilities in Poly(3-Methylthiophene) by an Electrochemical Doping. Synth. Met. 1998, 95 (1), 69–74. 10.1016/S0379-6779(98)00035-6.

[ref10] Paulsen B. D.; Frisbie C. D. Dependence of Conductivity on Charge Density and Electrochemical Potential in Polymer Semiconductors Gated with Ionic Liquids. J. Phys. Chem. C 2012, 116 (4), 3132–3141. 10.1021/jp2093934.

[ref11] Kang K.; Watanabe S.; Broch K.; Sepe A.; Brown A.; Nasrallah I.; Nikolka M.; Fei Z.; Heeney M.; Matsumoto D.; Marumoto K.; Tanaka H.; Kuroda S.; Sirringhaus H. 2D Coherent Charge Transport in Highly Ordered Conducting Polymers Doped by Solid State Diffusion. Nat. Mater. 2016, 15 (8), 896–902. 10.1038/nmat4634. 27159015

[ref12] Shimotani H.; Diguet G.; Iwasa Y. Direct Comparison of Field-Effect and Electrochemical Doping in Regioregular Poly(3-Hexylthiophene). Appl. Phys. Lett. 2005, 86 (2), 022104 10.1063/1.1850614.

[ref13] Liu J.; Shi Y.; Dong J.; Nugraha M. I.; Qiu X.; Su M.; Chiechi R. C.; Baran D.; Portale G.; Guo X.; Koster L. J. A. Overcoming Coulomb Interaction Improves Free-Charge Generation and Thermoelectric Properties for n-Doped Conjugated Polymers. ACS Energy Lett. 2019, 4 (7), 1556–1564. 10.1021/acsenergylett.9b00977.

[ref14] Jacobs I. E.; D’Avino G.; Lemaur V.; Lin Y.; Huang Y.; Chen C.; Harrelson T. F.; Wood W.; Spalek L. J.; Mustafa T.; O’Keefe C. A.; Ren X.; Simatos D.; Tjhe D.; Statz M.; Strzalka J. W.; Lee J.-K.; McCulloch I.; Fratini S.; Beljonne D.; Sirringhaus H. Structural and Dynamic Disorder, Not Ionic Trapping, Controls Charge Transport in Highly Doped Conducting Polymers. J. Am. Chem. Soc. 2022, 144 (7), 3005–3019. 10.1021/jacs.1c10651. 35157800 PMC8874922

[ref15] Rivnay J.; Inal S.; Collins B. A.; Sessolo M.; Stavrinidou E.; Strakosas X.; Tassone C.; Delongchamp D. M.; Malliaras G. G. Structural Control of Mixed Ionic and Electronic Transport in Conducting Polymers. Nat. Commun. 2016, 7 (1), 11287 10.1038/ncomms11287. 27090156 PMC4838877

[ref16] Bubnova O.; Khan Z. U.; Wang H.; Braun S.; Evans D. R.; Fabretto M.; Hojati-Talemi P.; Dagnelund D.; Arlin J.-B.; Geerts Y. H.; Desbief S.; Breiby D. W.; Andreasen J. W.; Lazzaroni R.; Chen W. M.; Zozoulenko I.; Fahlman M.; Murphy P. J.; Berggren M.; Crispin X. Semi-Metallic Polymers. Nat. Mater. 2014, 13 (2), 190–194. 10.1038/nmat3824. 24317188

[ref17] Liu J.; Qiu L.; Alessandri R.; Qiu X.; Portale G.; Dong J.; Talsma W.; Ye G.; Sengrian A. A.; Souza P. C. T.; Loi M. A.; Chiechi R. C.; Marrink S. J.; Hummelen J. C.; Koster L. J. A. Enhancing Molecular n-Type Doping of Donor–Acceptor Copolymers by Tailoring Side Chains. Adv. Mater. 2018, 30 (7), 1704630 10.1002/adma.201704630. 29325212

[ref18] Yang X.; Ye G.; Tran K.; Liu Y.; Cao J.; Dong J.; Portale G.; Liu J.; Zhang P.; Loi M. A.; Chiechi R. C.; Koster L. J. A. Impact of Oligo(Ethylene Glycol) Side Chains on the Thermoelectric Properties of Naphthalenediimide–Dialkoxybithiazole Polymers. ACS Mater. Lett. 2024, 6 (4), 1207–1215. 10.1021/acsmaterialslett.4c00068.

[ref19] Kuang Y.; Heester S.; Shao S.; Ye G.; Yao T.; Xie Z.; Koster L. J. A.; Liu J. Adjusting Molecular Weight Optimizes Electronic Transport of Extrinsically N-Type Doped Conjugated Polymer Incorporating Glycolated Side Chains. J. Mater. Chem. A 2024, 12 (8), 4866–4876. 10.1039/D3TA07188J.

[ref20] Liu J.; Craighero M.; Gupta V. K.; Scheunemann D.; Paleti S. H. K.; Järsvall E.; Kim Y.; Xu K.; Reparaz J. S.; Koster L. J. A.; Campoy-Quiles M.; Kemerink M.; Martinelli A.; Müller C. Electrically Programmed Doping Gradients Optimize the Thermoelectric Power Factor of a Conjugated Polymer. Adv. Funct. Mater. 2024, 34 (18), 2312549 10.1002/adfm.202312549.

[ref21] Efros A. L.; Shklovskii B. I. Coulomb Gap and Low Temperature Conductivity of Disordered Systems. J. Phys. C Solid State Phys. 1975, 8 (4), L49–L51. 10.1088/0022-3719/8/4/003.

[ref22] Efros A. L. Coulomb Gap in Disordered Systems. J. Phys. C Solid State Phys. 1976, 9 (11), 2021–2030. 10.1088/0022-3719/9/11/012.

[ref23] Arkhipov V. I.; Heremans P.; Emelianova E. V.; Adriaenssens G. J.; Bässler H. Charge Carrier Mobility in Doped Semiconducting Polymers. Appl. Phys. Lett. 2003, 82 (19), 3245–3247. 10.1063/1.1572965.

[ref24] Arkhipov V. I.; Heremans P.; Emelianova E. V.; Bässler H. Effect of Doping on the Density-of-States Distribution and Carrier Hopping in Disordered Organic Semiconductors. Phys. Rev. B 2005, 71 (4), 045214 10.1103/PhysRevB.71.045214.

[ref25] Arkhipov V. I.; Emelianova E. V.; Heremans P.; Bässler H. Analytic Model of Carrier Mobility in Doped Disordered Organic Semiconductors. Phys. Rev. B - Condens. Matter Mater. Phys. 2005, 72 (23), 235202 10.1103/PhysRevB.72.235202.

[ref26] Wang S.; Ha M.; Manno M.; Daniel Frisbie C.; Leighton C. Hopping Transport and the Hall Effect near the Insulator–Metal Transition in Electrochemically Gated Poly(3-Hexylthiophene) Transistors. Nat. Commun. 2012, 3 (1), 1210 10.1038/ncomms2213. 23169051

[ref27] Fediai A.; Symalla F.; Friederich P.; Wenzel W. Disorder Compensation Controls Doping Efficiency in Organic Semiconductors. Nat. Commun. 2019, 10 (1), 4547 10.1038/s41467-019-12526-6. 31591405 PMC6779899

[ref28] Koopmans M.; Leiviskä M. A. T.; Liu J.; Dong J.; Qiu L.; Hummelen J. C.; Portale G.; Heiber M. C.; Koster L. J. A. Electrical Conductivity of Doped Organic Semiconductors Limited by Carrier–Carrier Interactions. ACS Appl. Mater. Interfaces 2020, 12 (50), 56222–56230. 10.1021/acsami.0c15490. 33263385 PMC7747224

[ref29] Zhou J.; Zhou Y. C.; Zhao J. M.; Wu C. Q.; Ding X. M.; Hou X. Y. Carrier Density Dependence of Mobility in Organic Solids: A Monte Carlo Simulation. Phys. Rev. B 2007, 75 (15), 153201 10.1103/PhysRevB.75.153201.

[ref30] Liu F.; van Eersel H.; Xu B.; Wilbers J. G. E.; de Jong M. P.; van der Wiel W. G.; Bobbert P. A.; Coehoorn R. Effect of Coulomb Correlation on Charge Transport in Disordered Organic Semiconductors. Phys. Rev. B 2017, 96 (20), 205203 10.1103/PhysRevB.96.205203.

[ref31] Koopmans M.; Koster L. J. A. Carrier–Carrier Coulomb Interactions Reduce Power Factor in Organic Thermoelectrics. Appl. Phys. Lett. 2021, 119 (14), 143301 10.1063/5.0071208.

[ref32] Ge Y.; Liu R.; Shuai Z. Abnormal Seebeck Effect in Doped Conducting Polymers. Appl. Phys. Lett. 2021, 118 (12), 123301 10.1063/5.0043863.

[ref33] Kim E.-G.; Brédas J.-L. Electronic Evolution of Poly(3,4-Ethylenedioxythiophene) (PEDOT): From the Isolated Chain to the Pristine and Heavily Doped Crystals. J. Am. Chem. Soc. 2008, 130 (50), 16880–16889. 10.1021/ja806389b. 19053439

[ref34] Shi W.; Zhao T.; Xi J.; Wang D.; Shuai Z. Unravelling Doping Effects on PEDOT at the Molecular Level: From Geometry to Thermoelectric Transport Properties. J. Am. Chem. Soc. 2015, 137 (40), 12929–12938. 10.1021/jacs.5b06584. 26406937

[ref35] Zozoulenko I.; Singh A.; Singh S. K.; Gueskine V.; Crispin X.; Berggren M. Polarons, Bipolarons, And Absorption Spectroscopy of PEDOT. ACS Appl. Polym. Mater. 2019, 1 (1), 83–94. 10.1021/acsapm.8b00061.

[ref36] Yu S.; Wu H.-Y.; Lemaur V.; Kousseff C.; Beljonne D.; Fabiano S.; Nielsen C. Cation-Dependent Mixed Ionic-Electronic Transport in a Perylenediimide Small-Molecule Semiconductor. Angew. Chem., Int. Ed. 2024, e202410626 10.1002/anie.202410626. 39041291

[ref37] Wang D.; Shi W.; Chen J.; Xi J.; Shuai Z. Modeling Thermoelectric Transport in Organic Materials. Phys. Chem. Chem. Phys. 2012, 14 (48), 16505 10.1039/c2cp42710a. 23086525

[ref38] Shi W.; Wang D.; Shuai Z. High-Performance Organic Thermoelectric Materials: Theoretical Insights and Computational Design. Adv. Electron. Mater. 2019, 5 (11), 1800882 10.1002/aelm.201800882.

[ref39] Liu R.; Ge Y.; Wang D.; Shuai Z. Understanding the Temperature Dependence of the Seebeck Coefficient from First-Principles Band Structure Calculations for Organic Thermoelectric Materials. CCS Chem. 2021, 3 (10), 1477–1483. 10.31635/ccschem.021.202100813.

[ref40] Comin M.; Lemaur V.; Giunchi A.; Beljonne D.; Blase X.; D’Avino G. Doping of Semicrystalline Conjugated Polymers: Dopants within Alkyl Chains Do It Better. J. Mater. Chem. C 2022, 10 (37), 13815–13825. 10.1039/D2TC01115H.

[ref41] Muñoz W. A.; Singh S. K.; Franco-Gonzalez J. F.; Linares M.; Crispin X.; Zozoulenko I. V. Insulator to Semimetallic Transition in Conducting Polymers. Phys. Rev. B 2016, 94 (20), 205202 10.1103/PhysRevB.94.205202.

[ref42] Tjhe D. H. L.; Ren X.; Jacobs I. E.; D’Avino G.; Mustafa T. B. E.; Marsh T. G.; Zhang L.; Fu Y.; Mansour A. E.; Opitz A.; Huang Y.; Zhu W.; Unal A. H.; Hoek S.; Lemaur V.; Quarti C.; He Q.; Lee J.-K.; McCulloch I.; Heeney M.; Koch N.; Grey C. P.; Beljonne D.; Fratini S.; Sirringhaus H. Non-Equilibrium Transport in Polymer Mixed Ionic–Electronic Conductors at Ultrahigh Charge Densities. Nat. Mater. 2024, 10.1038/s41563-024-01953-6. PMC11599050 39060469

[ref43] Kang S. D.; Snyder G. J. Charge-Transport Model for Conducting Polymers. Nat. Mater. 2017, 16 (2), 252–257. 10.1038/nmat4784. 27842076

[ref44] Baranovskii S. D. Theoretical Description of Charge Transport in Disordered Organic Semiconductors. Phys. Status Solidi B 2014, 251 (3), 487–525. 10.1002/pssb.201350339. 25671376

[ref45] Paulsen B. D.; Fabiano S.; Rivnay J. Mixed Ionic-Electronic Transport in Polymers. Annu. Rev. Mater. Res. 2021, 51 (1), 73–99. 10.1146/annurev-matsci-080619-101319.

[ref46] Conjugated Conducting Polymers; Kiess H. G., Ed.; Cardona M., Fulde P., von Klitzing K., Queisser H.-J., Lotsch H. K. V., Lotsch H. K. V., Series Eds.; Springer Series in Solid-State Sciences; Springer Berlin Heidelberg: Berlin, Heidelberg, 1992; Vol. 102. 10.1007/978-3-642-46729-5.

[ref47] Bursill R. J.; Barford W. Electron-Lattice Relaxation, and Soliton Structures and Their Interactions in Polyenes. Phys. Rev. Lett. 1999, 82 (7), 1514–1517. 10.1103/PhysRevLett.82.1514.

[ref48] Barford W.; Bursill R. J.; Lavrentiev M. Y. Density-Matrix Renormalization-Group Calculations of Excited States of Linear Polyenes. Phys. Rev. B 2001, 63 (19), 195108 10.1103/PhysRevB.63.195108.

[ref49] Bursill R. J.; Barford W. Large-Scale Numerical Investigation of Excited States in Poly(Para-Phenylene). Phys. Rev. B - Condens. Matter Mater. Phys. 2002, 66 (20), 205112 10.1103/PhysRevB.66.205112.

[ref50] Race A.; Barford W.; Bursill R. J. Density Matrix Renormalization Calculations of the Relaxed Energies and Solitonic Structures of Polydiacetylene. Phys. Rev. B 2003, 67 (24), 245202 10.1103/PhysRevB.67.245202.

[ref51] Bursill R. J.; Barford W. Symmetry-Adapted Density Matrix Renormalization Group Calculations of the Primary Excited States of Poly(Para-Phenylene Vinylene). J. Chem. Phys. 2009, 130 (23), 234302 10.1063/1.3149536. 19548722

[ref52] Prodhan S.; Ramasesha S. Symmetrized Density Matrix Renormalization Group Algorithm for Low-Lying Excited States of Conjugated Carbon Systems: Application to 1,12-Benzoperylene and Polychrysene. Phys. Rev. B 2018, 97 (19), 195125 10.1103/PhysRevB.97.195125.

[ref53] Shahi M.; Le V. N.; Alarcon Espejo P.; Alsufyani M.; Kousseff C. J.; McCulloch I.; Paterson A. F. The Organic Electrochemical Transistor Conundrum When Reporting a Mixed Ionic–Electronic Transport Figure of Merit. Nat. Mater. 2024, 23, 2–8. 10.1038/s41563-023-01672-4. 37880535

[ref54] Chung J.; Khot A.; Savoie B. M.; Boudouris B. W. 100th Anniversary of Macromolecular Science Viewpoint: Recent Advances and Opportunities for Mixed Ion and Charge Conducting Polymers. ACS Macro Lett. 2020, 9 (5), 646–655. 10.1021/acsmacrolett.0c00037. 35648568

[ref55] Rolland N.; Franco-Gonzalez J. F.; Volpi R.; Linares M.; Zozoulenko I. V. Understanding Morphology-Mobility Dependence in PEDOT:Tos. Phys. Rev. Mater. 2018, 2 (4), 045605 10.1103/PhysRevMaterials.2.045605.

[ref56] Franco-Gonzalez J. F.; Rolland N.; Zozoulenko I. V. Substrate-Dependent Morphology and Its Effect on Electrical Mobility of Doped Poly(3,4-Ethylenedioxythiophene) (PEDOT) Thin Films. ACS Appl. Mater. Interfaces 2018, 10 (34), 29115–29126. 10.1021/acsami.8b08774. 30070463

[ref57] Khot A.; Savoie B. M. Top–Down Coarse-Grained Framework for Characterizing Mixed Conducting Polymers. Macromolecules 2021, 54 (10), 4889–4901. 10.1021/acs.macromol.1c00219.

[ref58] Makki H.; Troisi A. Morphology of Conducting Polymer Blends at the Interface of Conducting and Insulating Phases: Insight from PEDOT:PSS Atomistic Simulations. J. Mater. Chem. C 2022, 10 (42), 16126–16137. 10.1039/D2TC03158B. PMC9632246 36387833

[ref59] Landi A.; Reisjalali M.; Elliott J. D.; Matta M.; Carbone P.; Troisi A. Simulation of Polymeric Mixed Ionic and Electronic Conductors with a Combined Classical and Quantum Mechanical Model. J. Mater. Chem. C 2023, 11, 8062–8073. 10.1039/D2TC05103F. PMC10286221 37362027

[ref60] Prodhan S.; Manurung R.; Troisi A. From Monomer Sequence to Charge Mobility in Semiconductor Polymers via Model Reduction. Adv. Funct. Mater. 2023, 33 (36), 2303234 10.1002/adfm.202303234.

[ref61] Manurung R.; Troisi A. Screening Semiconducting Polymers to Discover Design Principles for Tuning Charge Carrier Mobility. J. Mater. Chem. C 2022, 10, 14319–14333. 10.1039/D2TC02527B. PMC9536249 36325475

[ref62] Lu T.; Chen F. Multiwfn: A Multifunctional Wavefunction Analyzer. J. Comput. Chem. 2012, 33 (5), 580–592. 10.1002/jcc.22885. 22162017

[ref63] Ohno K. Some Remarks on the Pariser-Parr-Pople Method. Theor. Chim Acta 1964, 2, 219–227. 10.1007/BF00528281.

[ref64] Klopman G. A Semiempirical Treatment of Molecular Structures. 11. Molecular Terms and Application to Diatomic Molecules. J. Am. Chem. Soc. 1964, 86, 4550–4557. 10.1021/ja01075a008.

[ref65] Prodhan S.; Mazumdar S.; Ramasesha S. Correlated Electronic Properties of a Graphene Nanoflake: Coronene. Molecules 2019, 24 (4), 730 10.3390/molecules24040730. 30781643 PMC6412552

[ref66] Balooch Qarai M.; Ghosh R.; Hestand N. J.; Spano F. C. Multipolaron Complexes in Conducting Polymers: The Importance of Hole–Hole Repulsion in Charge Delocalization. J. Phys. Chem. C 2023, 127 (13), 6414–6424. 10.1021/acs.jpcc.3c00289.

[ref67] Noriega R.; Rivnay J.; Vandewal K.; Koch F. P. V.; Stingelin N.; Smith P.; Toney M. F.; Salleo A. A General Relationship between Disorder, Aggregation and Charge Transport in Conjugated Polymers. Nat. Mater. 2013, 12 (11), 1038–1044. 10.1038/nmat3722. 23913173

[ref68] Fornari R. P.; Troisi A. Theory of Charge Hopping along a Disordered Polymer Chain. Phys. Chem. Chem. Phys. 2014, 16 (21), 9997–10007. 10.1039/c3cp54661f. 24481319

[ref69] Parris P. E.; Dunlap D. H.; Kenkre V. M. Energetic Disorder, Spatial Correlations, and the High-Field Mobility of Injected Charge Carriers in Organic Solids. Phys. Status Solidi B 2000, 218 (1), 47–53. 10.1002/(SICI)1521-3951(200003)218:1<47::AID-PSSB47>3.0.CO;2-T.

[ref70] Sirringhaus H. Device Physics of Solution-Processed Organic Field-Effect Transistors. Adv. Mater. 2005, 17 (20), 2411–2425. 10.1002/adma.200501152.

[ref71] Tessler N.; Preezant Y.; Rappaport N.; Roichman Y. Charge Transport in Disordered Organic Materials and Its Relevance to Thin-Film Devices: A Tutorial Review. Adv. Mater. 2009, 21 (27), 2741–2761. 10.1002/adma.200803541.

[ref72] Vukmirović N.; Wang L.-W. Charge Carrier Motion in Disordered Conjugated Polymers: A Multiscale Ab Initio Study. Nano Lett. 2009, 9 (12), 3996–4000. 10.1021/nl9021539. 19908900

[ref73] Frisch M. J.; Trucks G. W.; Schlegel H. B.; Scuseria G. E.; Robb M. A.; Cheeseman J. R.; Scalmani G.; Barone V.; Petersson G. A.; Nakatsuji H.; Li X.; Caricato M.; Marenich A. V.; Bloino J.; Janesko B. G.; Gomperts R.; Mennucci B.; Hratchian H. P.; Ortiz J. V.; Izmaylov A. F.; Sonnenberg J. L.; Williams-Young D.; Ding F.; Lipparini F.; Egidi F.; Goings J.; Peng B.; Petrone A.; Henderson T.; Ranasinghe D.; Zakrzewski V. G.; Gao J.; Rega N.; Zheng G.; Liang W.; Hada M.; Ehara M.; Toyota K.; Fukuda R.; Hasegawa J.; Ishida M.; Nakajima T.; Honda Y.; Kitao O.; Nakai H.; Vreven T.; Throssell K.; Montgomery, J. A.; Jr.; Peralta J. E.; Ogliaro F.; Bearpark M. J.; Heyd J. J.; Brothers E. N.; Kudin K. N.; Staroverov V. N.; Keith T. A.; Kobayashi R.; Normand J.; Raghavachari K.; Rendell A. P.; Burant J. C.; Iyengar S. S.; Tomasi J.; Cossi M.; Millam J. M.; Klene M.; Adamo C.; Cammi R.; Ochterski J. W.; Martin R. L.; Morokuma K.; Farkas O.; Foresman J. B.; Fox D. J. Gaussian 16, Rev. C.01.*;* Gaussian, Inc.: Wallingford CT, 2016.

[ref74] Barford W. Electronic and Optical Properties of Conjugated Polymers, Second ed.; International series of monographs on physics; Oxford University Press: Oxford, 2013.

[ref75] Virtanen P.; Gommers R.; Oliphant T. E.; Haberland M.; Reddy T.; Cournapeau D.; Burovski E.; Peterson P.; Weckesser W.; Bright J.; Van Der Walt S. J.; Brett M.; Wilson J.; Millman K. J.; Mayorov N.; Nelson A. R. J.; Jones E.; Kern R.; Larson E.; Carey C. J.; Polat İ.; Feng Y.; Moore E. W.; VanderPlas J.; Laxalde D.; Perktold J.; Cimrman R.; Henriksen I.; Quintero E. A.; Harris C. R.; Archibald A. M.; Ribeiro A. H.; Pedregosa F.; Van Mulbregt P.; Vijaykumar A.; Bardelli A. P.; Rothberg A.; Hilboll A.; Kloeckner A.; Scopatz A.; Lee A.; Rokem A.; Woods C. N.; Fulton C.; Masson C.; Häggström C.; Fitzgerald C.; Nicholson D. A.; Hagen D. R.; Pasechnik D. V.; Olivetti E.; Martin E.; Wieser E.; Silva F.; Lenders F.; Wilhelm F.; Young G.; Price G. A.; Ingold G.-L.; Allen G. E.; Lee G. R.; Audren H.; Probst I.; Dietrich J. P.; Silterra J.; Webber J. T.; Slavič J.; Nothman J.; Buchner J.; Kulick J.; Schönberger J. L.; De Miranda Cardoso J. V.; Reimer J.; Harrington J.; Rodríguez J. L. C.; Nunez-Iglesias J.; Kuczynski J.; Tritz K.; Thoma M.; Newville M.; Kümmerer M.; Bolingbroke M.; Tartre M.; Pak M.; Smith N. J.; Nowaczyk N.; Shebanov N.; Pavlyk O.; Brodtkorb P. A.; Lee P.; McGibbon R. T.; Feldbauer R.; Lewis S.; Tygier S.; Sievert S.; Vigna S.; Peterson S.; More S.; Pudlik T.; Oshima T.; Pingel T. J.; Robitaille T. P.; Spura T.; Jones T. R.; Cera T.; Leslie T.; Zito T.; Krauss T.; Upadhyay U.; Halchenko Y. O.; Vázquez-Baeza Y. SciPy 1.0: Fundamental Algorithms for Scientific Computing in Python. Nat. Methods 2020, 17 (3), 261–272. 10.1038/s41592-019-0686-2. 32015543 PMC7056644

[ref76] Mulliken R. S.; Rieke C. A.; Orloff D.; Orloff H. Overlap Integrals and Chemical Binding. J. Chem. Phys. 1949, 17 (5), 510–510. 10.1063/1.1747311.

[ref77] Chandross M.; Mazumdar S. Coulomb Interactions and Linear, Nonlinear, and Triplet Absorption in Poly(Para-Phenylenevinylene). Phys. Rev. B 1997, 55 (3), 1497–1504. 10.1103/PhysRevB.55.1497.

[ref78] Wang D.; Yu H.; Shi W.; Xu C. Chemical Doping of Organic and Coordination Polymers for Thermoelectric and Spintronic Applications: A Theoretical Understanding. Acc. Chem. Res. 2023, 56 (16), 2127–2138. 10.1021/acs.accounts.3c00091. 37432731

[ref79] Sony P.; Shukla A. A General Purpose Fortran 90 Electronic Structure Program for Conjugated Systems Using Pariser–Parr–Pople Model. Comput. Phys. Commun. 2010, 181 (4), 821–830. 10.1016/j.cpc.2009.12.015.

[ref80] Pople J. A.; Beveridge D. L. Approximate Molecular Orbital Theory; McGraw-Hill: New York, 1972.

[ref81] Fernández-Rossier J.; Palacios J. J. Magnetism in Graphene Nanoislands. Phys. Rev. Lett. 2007, 99 (17), 177204 10.1103/PhysRevLett.99.177204. 17995364

[ref82] Palacios J. J.; Fernández-Rossier J.; Brey L. Vacancy-Induced Magnetism in Graphene and Graphene Ribbons. Phys. Rev. B 2008, 77 (19), 195428 10.1103/PhysRevB.77.195428.

[ref83] Yazyev O. V. Magnetism in Disordered Graphene and Irradiated Graphite. Phys. Rev. Lett. 2008, 101 (3), 037203 10.1103/PhysRevLett.101.037203. 18764285

[ref84] Jung J.; MacDonald A. H. Carrier Density and Magnetism in Graphene Zigzag Nanoribbons. Phys. Rev. B 2009, 79 (23), 235433 10.1103/PhysRevB.79.235433.

[ref85] Szabo A.; Ostlund N. S. Modern Quantum Chemistry: Introduction to Advanced Electronic Structure Theory, 1. publ., unabr., unaltered republ. of the 1. ed., New York 1989.; Dover Publications: Mineola, NY, 1996.

[ref86] Thomas S.; Ramasesha S.; Hallberg K.; Garcia D. Fused Azulenes as Possible Organic Multiferroics. Phys. Rev. B 2012, 86 (18), 180403 10.1103/PhysRevB.86.180403.

[ref87] Goli V. M. L. D. P.; Prodhan S.; Mazumdar S.; Ramasesha S. Correlated Electronic Properties of Some Graphene Nanoribbons: A DMRG Study. Phys. Rev. B 2016, 94 (3), 035139 10.1103/PhysRevB.94.035139.

[ref88] Lieb E. H. Two Theorems on the Hubbard Model. Phys. Rev. Lett. 1989, 62 (10), 1201–1204. 10.1103/PhysRevLett.62.1201. 10039602

[ref89] Bässler H. Charge Transport in Disordered Organic Photoconductors a Monte Carlo Simulation Study. Phys. Status Solidi B 1993, 175 (1), 15–56. 10.1002/pssb.2221750102.

[ref90] Kim D.; Zozoulenko I. Why Is Pristine PEDOT Oxidized to 33%? A Density Functional Theory Study of Oxidative Polymerization Mechanism. J. Phys. Chem. B 2019, 123 (24), 5160–5167. 10.1021/acs.jpcb.9b01745. 31124678

[ref91] Kim D.; Franco-Gonzalez J. F.; Zozoulenko I. How Long Are Polymer Chains in Poly(3,4-Ethylenedioxythiophene):Tosylate Films? An Insight from Molecular Dynamics Simulations. J. Phys. Chem. B 2021, 125 (36), 10324–10334. 10.1021/acs.jpcb.1c04079. 34473507

