# Supplementary material for: Effective Model Reduction Scheme for the Electronic Structure of Highly Doped Semiconducting Polymers
Source: J Chem Theory Comput. 2024 Nov 4;20(22):10147–57. doi: 10.1021/acs.jctc.4c01131 (PMC11603604; doi:10.1021/acs.jctc.4c01131)
Supplement: Supplementary file 1 — ct4c01131_si_001.pdf [file ct4c01131_si_001.pdf]

Supporting Information: Effective Model Reduction Scheme for the Electronic Structure of Highly Doped Semiconducting Polymers

Suryoday Prodhan*† and Alessandro Troisi*

Department of Chemistry, University of Liverpool, Liverpool L69 3BX, UK

E-mail: suryoday.prodhan@liverpool.ac.uk; a.troisi@liverpool.ac.uk

† Current address: Department of Chemistry, Birla Institute of Technology and Science Pilani, Hyderabad Campus, Hyderabad 500078, India.

This supporting information contains:

S1. Electronic coupling between EDOT HOMOs
S2. Fitting of monomer hole density in singly-charged PEDOT-12 chain with or without $\epsilon_{chian\ end}$
S3. Fitting of monomer hole density in singly-charged PEDOT-12 chain with random counter-ion positions
S4. Parameter range for dual annealing scheme
S5. Computational workflow for optimization of single- and multiple-hole parameters
S6. Fitting of monomer hole density in multiple-hole PEDOT-12 chain with random counter-ion positions
S7. Derivation of the Fock matrix elements
S8. Self-consistent field (SCF) solutions within UHF calculations
S9. Optimal damping algorithm (ODA) for accelerating convergence of SCF solutions
S10. Torsional potential profile of PEDOT dimer
S11. PEDOT monomer HOMO energy distribution in PEDOT-PSS samples
S12. Distribution of ground state M_S in PEDOT chains of varying length and with varying number of holes
S13. Distribution of energy gaps in PEDOT chains of varying length and with varying number of holes

S1. Electronic coupling between EDOT HOMOs:

DFT calculations are done on geometry-optimized EDOT dimer with varying dihedral angles (θ) between the EDOT moieties and the electronic coupling between the HOMO orbitals of the EDOT moieties are calculated within the energy splitting in dimer method with Koopman's theorem approximation.[1] Within this approach, the absolute value of the electronic coupling is taken as,

$$|\tau| = \frac{E_{HOMO} - E_{HOMO-1}}{2} \qquad (S1)$$

where E_{HOMO} and E_{HOMO-1} are the energies of the HOMO and HOMO-1 levels of the neutral dimer respectively in the closed-shell configuration. Since HOMO orbital of EDOT is of π-symmetry, τ will be of opposite sign between $90° \leq \theta \leq 180°$ compared to $0° \leq \theta \leq 90°$ due to symmetry mismatch of the orbitals. Torsional angle dependence of τ is shown in Fig. S1 and it can be reproduced within a simple sinusoidal function $\tau = \tau_0 \cos\theta$ with root mean square error of fitting ~ 0.02 eV.[2]

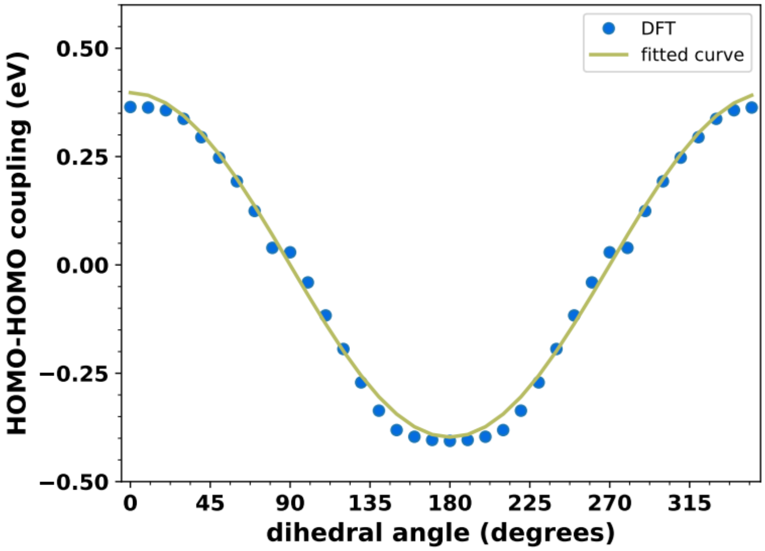

Figure S1: EDOT HOMO-HOMO electronic coupling as a function of dihedral angle and fitting of the coupling within a sinusoidal function.

S2. Fitting of monomer hole density in singly-charged PEDOT-12 chain with or without $\epsilon_{chian\ end}$:

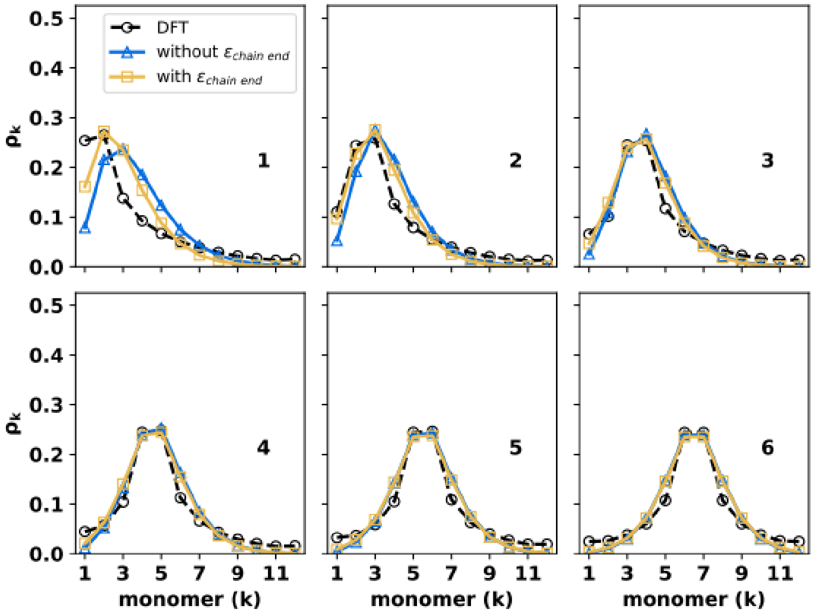

Figure S2: Monomer hole denisty profile in PEDOT-12 chains calculated within DFT (black broken, circle) and fitted within TB model without (blue solid, upper triangle) and with (orange solid, square) $\epsilon_{chain\ end}$ correction. The counter-ion positions are same as shown in Fig. 1 of the main manuscript. Better agreement betweem $\rho_{k,DFT}$ and $\rho_{k,TB}$ on including $\epsilon_{chain\ end}$, specifically when counter-ion remains towards PEDOT chain end, can be observed in the top left panel.

S3. Fitting of monomer hole density in singly-charged PEDOT-12 chain with random counter-ion positions:

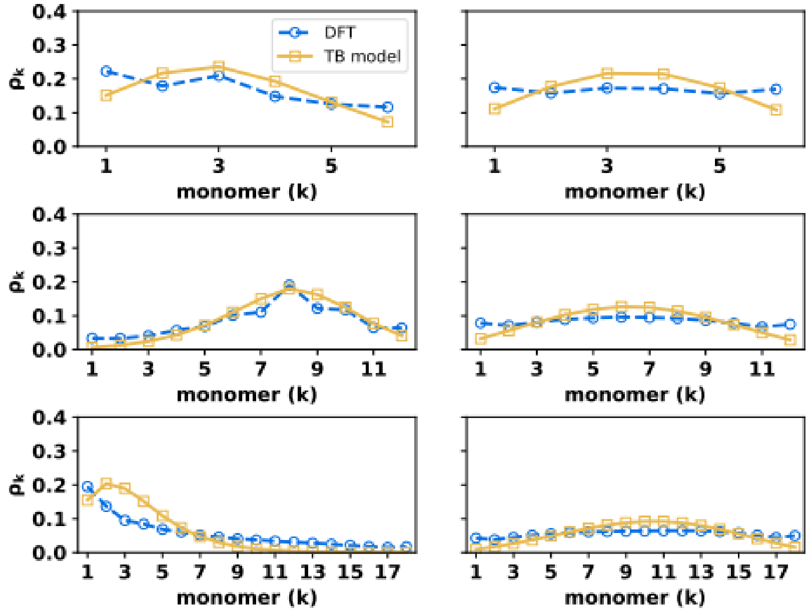

Figure S3: Monomer hole denisty profiles calculated within DFT (blue broken, circle) and fitted within TB model (orange solid, square) employing the optimized single-hole parameters for single-doped PEDOT-6 (top), PEDOT-12 (middle) and PEDOT-18 (bottom) chains with random counter-ion positions. The left panels represnt PEDOT chains with more localized holes while right panels represent significantly delocalized holes over the PEDOT chains.

S4. Parameter range for dual annealing scheme:

Table S1: Bounds of the single-hole and multiple-hole parameters optimized via dual annealing optimization algorithm. τ_0 range corresponds to electronic coupling values found in benchmark organic semiconducting polymers (see SI of Ref. 2) while U is searched within the range (0,10τ_0).

Parameter	Start value (eV)	End value (eV)
τ_0	0.01	10.0
$\epsilon_{chain\ end}$	0.0	10.0
U	0.0	61.5

S5. Computational workflow for optimization of single- and multiple-hole parameters:

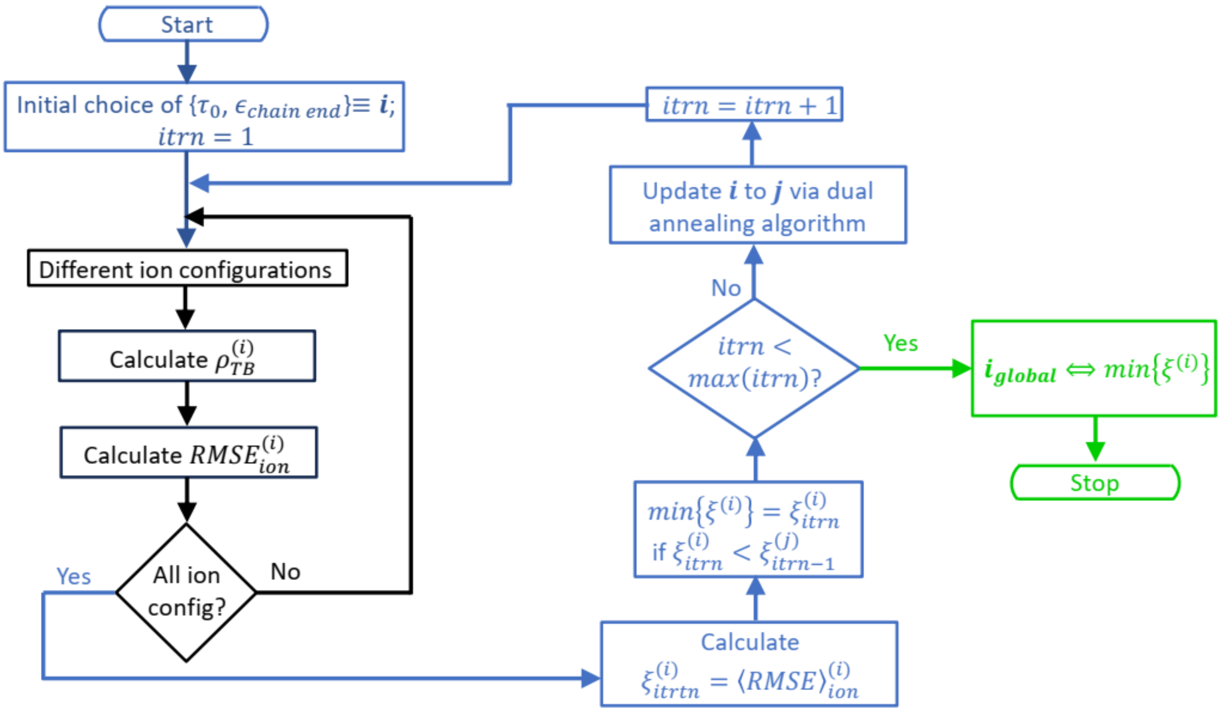

Figure S4: Computational workflow for determining the single-hole parameters (τ_0 and $\epsilon_{chain\ end}$). Loop shown in black is the inner loop while the one in blue is the outer loop. Dual annealing is carried out in the outer loop. Section in green represents exit of parameter optimization.

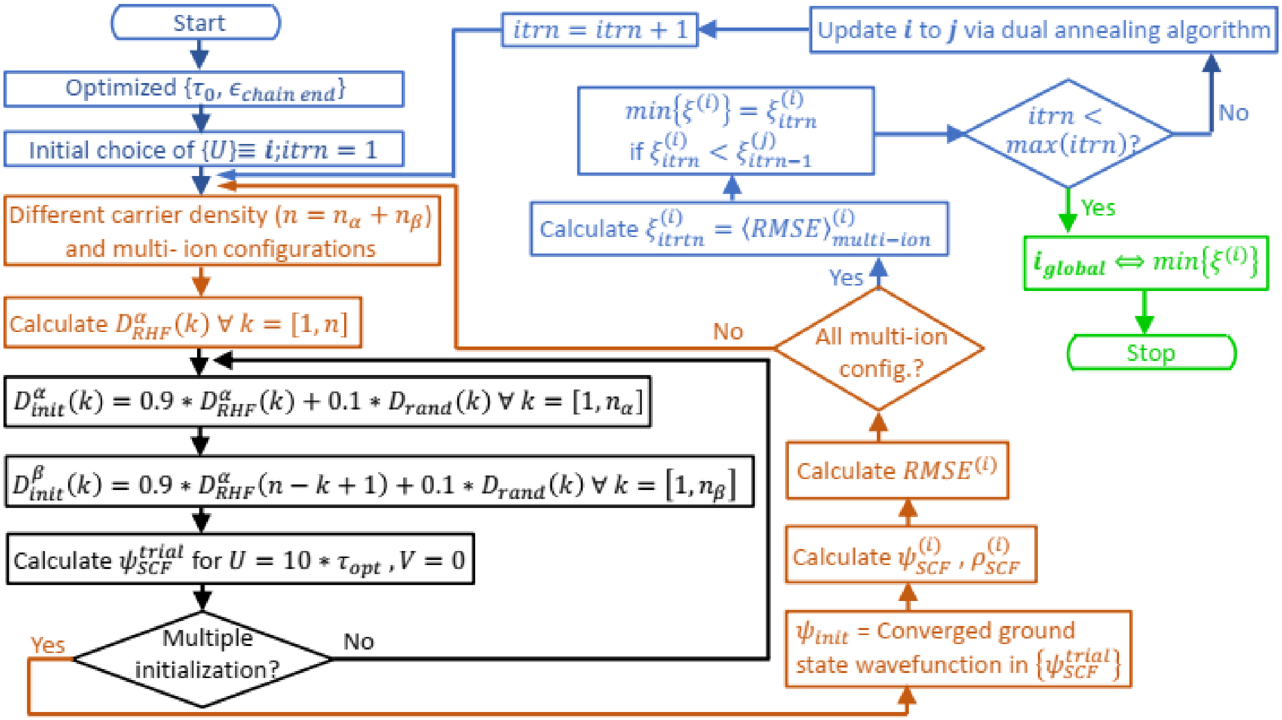

Figure S5: Computational workflow for determining the multiple-hole parameter (U). Loop shown in black is the innermost loop for generating the initial trial wavefunctions for SCF calculations while the one in blue is the outermost loop. Dual annealing is carried out in the outermost loop while fitting of ρ_k is carried out in the loop shown in orange. Section in green represents exit of parameter optimization.

S6. Fitting of monomer hole density in multiple-hole PEDOT-12 chain with random counter-ion positions:

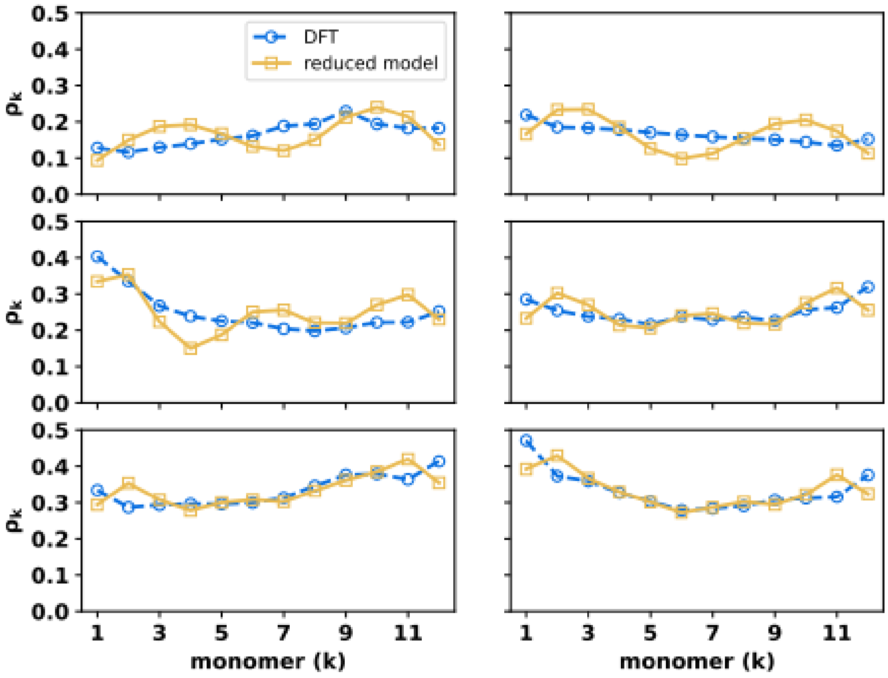

Figure S6: Representative monomer hole denisty profiles calculated within DFT (blue broken, circle) and fitted within the reduced model Hamiltonian (Eq. 1 in the main manuscript) (orange solid, square) for PEDOT-12 chains with multiple holes. The hole density profiles are shown for PEDOT-12 with 2 holes (top panels), 3 holes (middle panels) and 4 holes (bottom panels) on each chain.

S7. Derivation of the Fock matrix elements:

The People-Nesbet equations[3] within the unrestricted Hartree-Fock (UHF) scheme is given by,

$$\sum_j \left(F_{ij}^{\alpha(\beta)} - E_\mu^{\alpha(\beta)} S_{ij}\right) C_{j\mu}^{\alpha(\beta)} = 0 \qquad (S2)$$

where $E_\mu^{\alpha(\beta)}$ is the UHF eigenvalue of the μ-th $\alpha(\beta)$-spin orbital (ψ_μ^α and ψ_μ^β) and $\left\{C_{i\mu}^{\alpha(\beta)}\right\}$ are the coefficients of corresponding $\alpha(\beta)$-spin orbital in the monomer HOMO-orbital basis $\{\phi_i\}$; in present study, we consider $C_{i\mu}^{\alpha(\beta)}$ is real.

$$\psi_\mu^{\alpha(\beta)} = \sum_i C_{i\mu}^{\alpha(\beta)} \phi_i \qquad (S3)$$

$F_{ij}^{\alpha(\beta)}$ is the matrix element of the Fock matrix for $\alpha(\beta)$-spin while S_{ij} is the overlap matrix between basis states ϕ_i and ϕ_j. In the present case, we assume that $\{\phi_i\}$ corresponds to an orthonormal basis set and the overlap between neighboring monomer HOMO orbitals is small ($S_{ij} \approx \delta_{ij}$). Following this approximation one can convert Eq. S3 into,

$$\sum_j \left(F_{ij}^{\alpha(\beta)} - E_\mu^{\alpha(\beta)} \delta_{ij}\right) C_{j\mu}^{\alpha(\beta)} = 0 \qquad (S4)$$

$$\sum_j F_{ij}^{\alpha(\beta)} C_{j\mu}^{\alpha(\beta)} = E_\mu^{\alpha(\beta)} C_{i\mu}^{\alpha(\beta)} \qquad (S5)$$

The Fock matrix elements for $\alpha(\beta)$-spin is given by,

$$F_{ij}^{\alpha(\beta)} = h_{ij} + \sum_k \sum_l \left[P_{kl}(ij|kl) - P_{kl}^{\alpha(\beta)}(il|kj)\right] \qquad (S6)$$

where h_{ij} is the one-particle Hamiltonian matrix element,

$$h_{ij} = \int dr_1 \phi_i(1) h_1 \phi_j(1) \qquad (S7)$$

and $(ij|kl)$ are the four-center two-partricle integrals,

$$(ij|kl) = \int dr_1 \int dr_2 \phi_i(1)\phi_j(1) \frac{1}{|r_1 - r_2|} \phi_k(2)\phi_l(2) \qquad (S8).$$

The first term within the summation in Eq. S6 is the Coulomb term of the Fock operator which contains interaction terms between both α- and β-spins. On the other hand, the second term within the summation represents the exchange term of the Fock operator which has contributions only from orbitals with same spin.

$P^{\alpha(\beta)}$ is the density matrix for $\alpha(\beta)$-spin corresponding to $N_\alpha(N_\beta)$ particles while P is the total density matrix of the N_h number of quantum particles ($N_\alpha + N_\beta = N_h$).

$$P_{ij}^{\alpha(\beta)} = \sum_{\mu=1}^{N_{\alpha(\beta)}} C_{i\mu}^{\alpha(\beta)} C_{j\mu}^{\alpha(\beta)} \qquad (S9)$$

$$P_{ij} = P_{ij}^{\alpha} + P_{ij}^{\beta} \qquad (S10)$$

The effective model Hamiltonian, given in Eq. 1 in the main manuscript is semi-empirical and implicitly maintains complete neglect of differential overlap (CNDO) approximation so that,

$$(ij|kl) = (ii|kk)\delta_{ij}\delta_{kl} \qquad (S11).$$

The surviving two-center two-particle integrals are,

$$V_{ij} = (ii|jj) \qquad (S12)$$

$$U = V_{ii} = (ii|ii) \qquad (S13)$$

Applying CNDO approximation to Fock matrix element expressions (Eq. S6) and using Eq. S12 and S13, we reproduce Eq. 7 and 8 in the main manuscript.

$$\begin{aligned} F_{ii}^{\alpha(\beta)} &= h_{ii} + \sum_{k}\sum_{l}\left[P_{kl}(ii|kl)\delta_{kl} - P_{kl}^{\alpha(\beta)}(il|ki)\delta_{il}\delta_{ki}\right] \\ &= h_{ii} + \sum_{k} P_{kk}(ii|kk) - P_{ii}^{\alpha(\beta)}(ii|ii) \\ &= h_{ii} + \sum_{j} P_{jj}V_{ij} - P_{ii}^{\alpha(\beta)}U \qquad (S14) \end{aligned}$$

$$\begin{aligned} F_{ij}^{\alpha(\beta)} &= h_{ij} + \sum_{k}\sum_{l}\left[P_{kl}(ij|kl)\delta_{ij}\delta_{kl} - P_{kl}^{\alpha(\beta)}(il|kj)\delta_{il}\delta_{kj}\right] \\ &= h_{ij} - P_{ji}^{\alpha(\beta)}(ii|jj) \\ &= h_{ij} - P_{ij}^{\alpha(\beta)}V_{ij} \qquad (S15) \end{aligned}$$

In Eq. S15, we employ the symmetry of the density matrices ($P_{ji} = P_{ij}$) to obtain the final expression.

S8. Self-consistent field (SCF) solutions within UHF calculations:

Within the UHF formalism, the wavefunctions are determined by iteratively solving the Pople-Nesbet equations (Eq. S2). However, if $N_\alpha = N_\beta$, two possible independent solutions of the Pople-Nesbet equations are possible and an initial guess of $P^{\alpha}_{trial} = P^{\beta}_{trial}$ will always results to the restricted Hartree-Fock (RHF) wavefunctions which may be of higher-energy compared to the alternative solution.[4] Therefore, to obtain the non-trivial solution of the Pople-Nesbet equations, it is imperative that an initial guess of $P^{\alpha}_{trial} \neq P^{\beta}_{trial}$ is used and for that purpose, we use partial randomization of RHF solutions.

We first compute the RHF wavefunctions ($\psi_{\mu,RHF}$) of a one-dimensional chain with N sites corresponding to the Hamiltonian $H^{RHF} = \left[H_{ij}\right]_{N\times N}$ where $H_{ii} = 0$ and $H_{i,i+1} = \tau_0 \cos\theta_{i,i+1}$, τ_0 and $\theta_{i,i+1}$ defined in the main manuscript. Employing the RHF wavefunctions, the initial trial wavefunctions for UHF calculations are generated as,

$$\psi^{\alpha}_{\mu,trial} = (1-\alpha_{rand})\psi_{\mu,RHF} + \alpha_{rand}\psi_{rand} \qquad (S16)$$

$$\psi^{\beta}_{\mu,trial} = (1-\alpha_{rand})\psi_{N-\mu+1,RHF} + \alpha_{rand}\psi_{rand} \qquad (S17)$$

where ψ_{rand} is a random vector with elements $0 \leq \psi_{i,rand} < 1$ and $\alpha_{rand} = 0.1$ is the extent of randomization, followed by orthonormalization of $\left\{\psi^{\alpha(\beta)}_{\mu,trial}\right\}$ and calculation of $P^{\alpha(\beta)}_{trial}$.

Since the solutions of the Pople-Nesbet equations are derived through SCF iteration procedure we employ three strategies (listed below) for accelerating the SCF convergence. In the present study, we set the convergence threshold of 10^{-8} eV for SCF energy and 10^{-4} eV in corresponding orbital energies between successive iterations.

(1) Initial trial density matrices $P^{\alpha(\beta)}_{trial}$ for SCF calculations of the model Hamiltonian (Eq. 1, main manuscript) are not chosen randomly but set forward from the SCF solutions of a trial Hamiltonian

$$H_{trial} = \sum_{k,\sigma} \tau_{k,k+1}\left[c^{\dagger}_{k,\sigma}c_{k+1,\sigma} + c^{\dagger}_{k+1,\sigma}c_{k,\sigma}\right] + \sum_{k,a} W_{ka}(R_{ka})\hat{n}_k + \sum_{k} \frac{U_{trial}}{2}\hat{n}_k(\hat{n}_k - 1) \qquad (S18).$$

$U_{trial} = 10\tau_0$ represents a system where two holes of opposite spins are very unlikely to occupy the same site due to large energy cost. The system is characteristically similar to H_2 molecule where the two hydrogen atoms are separated far away from the equilibrium distance and the UHF energy is lower compared to RHF energy.[4] As the correct SCF energy and wavefunction can be retraced back on lowering the separation between the hydrogen atoms, the solutions of H_{trial} is adopted to recover the SCF solution of the final model Hamiltonian (Eq. 1, main manuscript). For accuracy, we determine the SCF solutions of H_{trial} initialized multiple times employing Eq. S16 and S17 (100 random initializations, shown as loop in black in Fig. S5), and the minimum energy solution is chosen to compute $P^{\alpha(\beta)}_{trial}$.

(2) For the first 100 cycles of the iterations, we employ simple damping algorithm (SDA)[5] which mixes the Fock matrices at $(n+1)$-th iteration, calculated from the density matrices obtained at n-th iteration, with the Fock matrices of previous iterations.

$$\tilde{F}^{\alpha(\beta)}_{n+1} = (1-\lambda_{SD})\tilde{F}^{\alpha(\beta)}_{n} + \lambda_{SD}F^{\alpha(\beta)}_{n+1} \qquad (S19)$$

$$F^{\alpha(\beta)}_{n+1} = F\left(P^{\alpha(\beta)}_{n}\right) \qquad (S20)$$

In the present calculations, we set $\lambda_{SD} = 0.90$. The mixed Fock matrices $\tilde{F}^{\alpha(\beta)}_{n+1}$ are employed to compute the density matrices $P^{\alpha(\beta)}_{n+1}$ at the $(n+1)$-th iteration and simple damping in the above way (Eq. S19 and S20) retains idempotency of the density matrices.[5]

(3) If the convergence criteria given above are not met within SDA formalism, optimal damping algorithm (ODA),[6,7] developed by Cancès *et al.* is employed considering the density matrices obtained at the last iteration of SDA as the initial, trial density matrices for ODA. Brief details as well as the algorithmic flow of ODA is given in the next section. A maximum of 1000 iterations are considered within the ODA for achieving SCF convergence.

S9. Optimal damping algorithm (ODA) for accelerating convergence of SCF solutions:

ODA is a variation of the relaxed constraints algorithms (RCA) which is a direct minimization procedure of E^{UHF} on the convex set $\tilde{\Pi}_n^{\alpha(\beta)} = \{\tilde{P}^{\alpha(\beta)}, \tilde{P}^{\alpha(\beta),*} = \tilde{P}^{\alpha(\beta)}, Tr(\tilde{P}^{\alpha(\beta)}) = N_{\alpha(\beta)}, \tilde{P}^{\alpha(\beta)}\tilde{P}^{\alpha(\beta)} \leq \tilde{P}^{\alpha(\beta)}\}$ obtained from $\Pi_n^{\alpha(\beta)} = \{P^{\alpha(\beta)}, P^{\alpha(\beta),*} = P^{\alpha(\beta)}, Tr(P^{\alpha(\beta)}) = N_{\alpha(\beta)}, P^{\alpha(\beta)}P^{\alpha(\beta)} = P^{\alpha(\beta)}\}$ by relaxing the idempotency constraint of $P^{\alpha(\beta)}$.[6] The technique differs from the direct inversion of the iterative subspace (DIIS)[8,9] technique since the intrinsic algorithm implemented in the latter does not force convergence in energy[10] and indeed, does fail at instances far away from convergence.

The derivative of E^{UHF} directed from a relaxed density matrix $\tilde{P}^{\alpha(\beta)} \in \tilde{\Pi}_n^{\alpha(\beta)}$ towards any other density matrix $P'^{\alpha(\beta)} \in \Pi_n^{\alpha(\beta)}$ is given by,

$$s_{\tilde{P}^{\alpha(\beta)} \to P'^{\alpha(\beta)}} = Tr\left[F\left(\tilde{P}^{\alpha(\beta)}\right)\left(P'^{\alpha(\beta)} - \tilde{P}^{\alpha(\beta)}\right)\right] \qquad (S21),$$

and the steepest-descent direction is derived by extracting the minimal density matrix $P^{\alpha(\beta)} \in \Pi_n^{\alpha(\beta)}$ via minimizing

$$P^{\alpha(\beta)} = arg\left(min\left(s_{\tilde{P}^{\alpha(\beta)} \to P'^{\alpha(\beta)}}, P'^{\alpha(\beta)} \in \Pi_n^{\alpha(\beta)}\right)\right) \qquad (S22).$$

The steepest-descent direction is also the direction obtained by Aufbau principle (choosing the set of orthonormal molecular orbitals corresponding to the smallest eigenvalues).[11] Within the ODA algorithm, these attributes are employed conversely *i.e.,* starting from a particular $\tilde{P}_n^{\alpha(\beta)}$, the corresponding Fock matrix is constructed and diagonalized, followed by construction of $P_{n+1}^{\alpha(\beta)}$ employing aufbau principle, and then the relaxed density matrix $\tilde{P}_{n+1}^{\alpha(\beta)}$ corresponding to the lowest E^{UHF} is searched over the line segment joining $\tilde{P}_n^{\alpha(\beta)}$ and $P_{n+1}^{\alpha(\beta)}$. $P_n^{\alpha(\beta)} = \tilde{P}_n^{\alpha(\beta)} = P_{n+1}^{\alpha(\beta)} = \tilde{P}_{n+1}^{\alpha(\beta)} = \cdots$ on achieving convergence since the slope $s_{\tilde{P}_n^{\alpha(\beta)} \to P_{n+1}^{\alpha(\beta)}}$ becomes zero while $s_{\tilde{P}_n^{\alpha(\beta)} \to P_{n+1}^{\alpha(\beta)}}$ is a negative quantity before convergence is achieved. Minimization of E^{UHF} on the line segment $seg\left[\tilde{P}_n^{\alpha(\beta)}, P_{n+1}^{\alpha(\beta)}\right]$ is particularly convenient within the HF setting since energy is quadratic with respect to the density matrices.[6]

We employ the alternate version of ODA developed by Yamamoto and coworkers for UHF and the basic formulation is given below;[12] for simplicity, we only describe how to derive the optimal P_{n+1}^{β} at the $(n+1)$-th iteration keeping a strict P_{n+1}^{α} – the procedure is similar to calculate the optimal P_{n+1}^{α}.

P_{n+1}^{β} is updated by constructing the relaxed β-spin density matrix $\tilde{P}_{n+1}^{\beta}$ with the mixing constant $\lambda_\beta \in [0,1]$,

$$\tilde{P}_{n+1}^{\beta} = (1-\lambda_\beta)\tilde{P}_n^{\beta} + \lambda_\beta P_{n+1}^{\beta} = \tilde{P}_n^{\beta} + \lambda_\beta\left(P_{n+1}^{\beta} - \tilde{P}_n^{\beta}\right) = \tilde{P}_n^{\beta} + \lambda_\beta \Delta P_{n+1}^{\beta} \qquad (S23).$$

The Fock operator for β-spin calculated at the $(n+1)$-th iteration is described by,

$$F_{n+1}^{\beta} = F\left(P_{n+1}^{\beta}, P_{n+1}^{\alpha}\right) = h + G\left(P_{n+1}^{\beta}\right) + J(P_{n+1}^{\alpha}) \qquad (S24)$$

where $G(P) = J(P) - K(P)$, J and K being the Coulomb and exchange terms of the Fock operator. We can obtain similar relaxed Fock operators ($\tilde{F}_{n+1}^{\beta}$) considering relaxed density matrix $\tilde{P}_{n+1}^{\beta}$ in conjugation with P_{n+1}^{α}.

$$\begin{aligned}
\tilde{F}_{n+1}^{\beta} &= F\left(\tilde{P}_{n+1}^{\beta}, P_{n+1}^{\alpha}\right) \\
&= h + G\left(\tilde{P}_{n+1}^{\beta}\right) + J(P_{n+1}^{\alpha}) \\
&= h + G\left((1-\lambda_\beta)\tilde{P}_n^{\beta} + \lambda_\beta P_{n+1}^{\beta}\right) + J(P_{n+1}^{\alpha}) \\
&= h + G\left((1-\lambda_\beta)\tilde{P}_n^{\beta}\right) + G\left(\lambda_\beta P_{n+1}^{\beta}\right) + J(P_{n+1}^{\alpha})
\end{aligned}$$

$$= (1-\lambda_\beta)\left[h + G\left(\tilde{P}_n^\beta\right) + J(P_n^\alpha)\right] + \lambda_\beta\left[h + G\left(P_{n+1}^\beta\right) + J(P_{n+1}^\alpha)\right] + (1-\lambda_\beta)[J(P_{n+1}^\alpha) - J(P_n^\alpha)]$$
$$= (1-\lambda_\beta)\tilde{F}_n^\beta + \lambda_\beta F_{n+1}^\beta + (1-\lambda_\beta)[J_{n+1}^\alpha - J_n^\alpha] \qquad (S25)$$

The optimal value of the parameter λ_β is derived by minimization of UHF energy $E^{UHF}\left(P_{n+1}^\alpha, \tilde{P}_{n+1}^\beta\right)$ *i.e.,* by setting

$$\frac{dE^{UHF}}{d\lambda_\beta} = 0 \qquad (S26).$$

The UHF energy expression with relaxed β-spin density matrix and fixed α-spin density matrix is given by,

$$E^{UHF}\left(P_{n+1}^\alpha, \tilde{P}_{n+1}^\beta\right)$$
$$= Tr\left[h\left[P_{n+1}^\alpha + \tilde{P}_{n+1}^\beta\right] + \frac{1}{2}G(P_{n+1}^\alpha)P_{n+1}^\alpha + \frac{1}{2}G\left(\tilde{P}_{n+1}^\beta\right)\tilde{P}_{n+1}^\beta + J\left(\tilde{P}_{n+1}^\beta\right)P_{n+1}^\alpha\right]$$
$$= Tr\left[h\left[P_{n+1}^\alpha + \tilde{P}_n^\beta + \lambda_\beta\left[P_{n+1}^\beta - \tilde{P}_n^\beta\right]\right] + \frac{1}{2}G(P_{n+1}^\alpha)P_{n+1}^\alpha + \frac{1}{2}G\left(\tilde{P}_n^\beta + \lambda_\beta\left[P_{n+1}^\beta - \tilde{P}_n^\beta\right]\right)\left[\tilde{P}_n^\beta + \lambda_\beta\left[P_{n+1}^\beta - \tilde{P}_n^\beta\right]\right]\right.$$
$$\left. + J\left(\tilde{P}_n^\beta + \lambda_\beta\left[P_{n+1}^\beta - \tilde{P}_n^\beta\right]\right)P_{n+1}^\alpha\right]$$
$$= Tr\left[h\left[P_{n+1}^\alpha + \tilde{P}_n^\beta\right] + \frac{1}{2}G(P_{n+1}^\alpha)P_{n+1}^\alpha + \frac{1}{2}G\left(\tilde{P}_n^\beta\right)\tilde{P}_n^\beta + J\left(\tilde{P}_n^\beta\right)P_{n+1}^\alpha\right]$$
$$+ \lambda_\beta Tr\left[h\left[P_{n+1}^\beta - \tilde{P}_n^\beta\right] + \frac{1}{2}G\left(P_{n+1}^\beta - \tilde{P}_n^\beta\right)\tilde{P}_n^\beta + \frac{1}{2}G\left(\tilde{P}_n^\beta\right)\left[P_{n+1}^\beta - \tilde{P}_n^\beta\right] + J\left(P_{n+1}^\beta - \tilde{P}_n^\beta\right)P_{n+1}^\alpha\right]$$
$$+ (\lambda_\beta)^2 Tr\left[\frac{1}{2}G\left(P_{n+1}^\beta - \tilde{P}_n^\beta\right)\left[P_{n+1}^\beta - \tilde{P}_n^\beta\right]\right]$$
$$= E^{UHF}\left(P_{n+1}^\alpha, \tilde{P}_n^\beta\right) + \lambda_\beta Tr\left[\left[h + G\left(\tilde{P}_n^\beta\right) + J(P_{n+1}^\alpha)\right]\Delta P_{n+1}^\beta\right] + (\lambda_\beta)^2 Tr\left[\frac{1}{2}G\left(P_{n+1}^\beta - \tilde{P}_n^\beta\right)\Delta P_{n+1}^\beta\right] \qquad (S27)$$

To derive the simplified form of Eq. S27, we employ Eq. S23 and the following identities,[6]

$$Tr\left[G\left(P_j^\beta\right)P_i^\beta\right] = Tr\left[G\left(P_i^\beta\right)P_j^\beta\right] \qquad (S28)$$
$$Tr\left[J\left(P_j^\beta\right)P_i^\alpha\right] = Tr\left[J(P_i^\alpha)P_j^\beta\right] \qquad (S29).$$

On further simplification of Eq. S27, we obtain

$$E^{UHF}\left(P_{n+1}^\alpha, \tilde{P}_{n+1}^\beta\right)$$
$$= E^{UHF}\left(P_{n+1}^\alpha, \tilde{P}_n^\beta\right) + \lambda_\beta Tr\left[F\left(\tilde{P}_n^\beta, P_{n+1}^\alpha\right)\Delta P_{n+1}^\beta\right] + (\lambda_\beta)^2 Tr\left[\frac{1}{2}G\left(P_{n+1}^\beta - \tilde{P}_n^\beta\right)\Delta P_{n+1}^\beta\right]$$
$$= E^{UHF}\left(P_{n+1}^\alpha, \tilde{P}_n^\beta\right) + \lambda_\beta Tr\left[F\left(\tilde{P}_n^\beta, P_{n+1}^\alpha\right)\Delta P_{n+1}^\beta\right] + (\lambda_\beta)^2 Tr\left[\frac{1}{2}\left[G\left(P_{n+1}^\beta\right) - G\left(\tilde{P}_n^\beta\right)\right]\Delta P_{n+1}^\beta\right]$$
$$= E^{UHF}\left(P_{n+1}^\alpha, \tilde{P}_n^\beta\right) + \lambda_\beta Tr\left[F\left(\tilde{P}_n^\beta, P_{n+1}^\alpha\right)\Delta P_{n+1}^\beta\right]$$
$$+ (\lambda_\beta)^2 Tr\left[\frac{1}{2}\left[h + G\left(P_{n+1}^\beta\right) + J(P_{n+1}^\alpha) - h - G\left(\tilde{P}_n^\beta\right) - J(P_{n+1}^\alpha)\right]\Delta P_{n+1}^\beta\right]$$
$$= E^{UHF}\left(P_{n+1}^\alpha, \tilde{P}_n^\beta\right) + \lambda_\beta Tr\left[F\left(\tilde{P}_n^\beta, P_{n+1}^\alpha\right)\Delta P_{n+1}^\beta\right] + (\lambda_\beta)^2\frac{1}{2}Tr\left[\left[F\left(P_{n+1}^\beta, P_{n+1}^\alpha\right) - F\left(\tilde{P}_n^\beta, P_{n+1}^\alpha\right)\right]\Delta P_{n+1}^\beta\right]$$
$$= E^{UHF}\left(P_{n+1}^\alpha, \tilde{P}_n^\beta\right) + \lambda_\beta s_\beta + c_\beta(\lambda_\beta)^2 \qquad (S30)$$

where,

$$s_\beta = Tr\left[F\left(\tilde{P}_n^\beta, P_{n+1}^\alpha\right)\Delta P_{n+1}^\beta\right]$$
$$= Tr\left[\left[h + G\left(\tilde{P}_n^\beta\right) + J(P_{n+1}^\alpha)\right]\Delta P_{n+1}^\beta\right]$$

$$= Tr\left[\left[h + G\left(\tilde{P}_n^{\beta}\right) + J(P_n^{\alpha}) + J(P_{n+1}^{\alpha}) - J(P_n^{\alpha})\right]\Delta P_{n+1}^{\beta}\right]$$
$$= Tr\left[\left[F\left(\tilde{P}_n^{\beta}, P_n^{\alpha}\right) + J(P_{n+1}^{\alpha}) - J(P_n^{\alpha})\right]\Delta P_{n+1}^{\beta}\right]$$
$$= Tr\left[\left[\tilde{F}_n^{\beta} + J_{n+1}^{\alpha} - J_n^{\alpha}\right]\Delta P_{n+1}^{\beta}\right] \qquad (S31),$$

and,

$$c_{\beta} = \frac{1}{2}Tr\left[\left[F\left(P_{n+1}^{\beta}, P_{n+1}^{\alpha}\right) - F\left(\tilde{P}_n^{\beta}, P_{n+1}^{\alpha}\right)\right]\Delta P_{n+1}^{\beta}\right]$$
$$= \frac{1}{2}Tr\left[\left[F_{n+1}^{\beta} - \tilde{F}_n^{\beta} - J_{n+1}^{\alpha} + J_n^{\alpha}\right]\Delta P_{n+1}^{\beta}\right] \qquad (S32).$$

Therefore, the minimization condition of E^{UHF} (Eq. S26) becomes

$$s_{\beta} + 2\lambda_{\beta}c_{\beta} = 0; \lambda_{\beta} \in [0,1] \qquad (S33).$$

The optimal value of λ_{β} is given by

$$\lambda_{\beta} = \begin{cases} 1 \quad if\ |c_{\beta}| \leq \dfrac{|s_{\beta}|}{2}\ or\ s_{\beta}c_{\beta} \geq 0 \\ -\dfrac{s_{\beta}}{2c_{\beta}} \quad otherwise \end{cases} \qquad (S34),$$

which differs slightly from that derived for RHF in Ref. 6 since the simple steepest-descent strategy is not valid due to stepwise coupling with density matrices of opposite spin.

Algorithmic flow:

1. Initialization: Initial guess of P_0^{α} and P_0^{β} are chosen (in the present study using the density matrices obtained at the final iteration of SDA) and $F_0^{\alpha} = F\left(P_0^{\alpha}, P_0^{\beta}\right)$, $F_0^{\beta} = F\left(P_0^{\beta}, P_0^{\alpha}\right)$, $J_0^{\alpha} = J(P_0^{\alpha})$, $J_0^{\beta} = J\left(P_0^{\beta}\right)$ and $E_0^{UHF} = E^{UHF}\left(P_0^{\alpha}, P_0^{\beta}\right)$ are computed. We also set $\tilde{P}_0^{\alpha} = P_0^{\alpha}$, $\tilde{F}_0^{\alpha} = F_0^{\alpha}$ while $\tilde{P}_{-1}^{\beta}$ and $\tilde{F}_{-1}^{\beta}$ are set to the respective P^{β} and F^{β} computed at the penultimate iteration of SDA. $\Delta P_k^{\alpha(\beta)} = 0$ is set while the iteration number k is set to zero.

2. Iteration:

(a) $\tilde{F}_k^{\alpha}$ is diagonalized and P_{k+1}^{α} is calculated within the Aufbau principle.

(b) $J_{k+1}^{\alpha} = J(P_{k+1}^{\alpha})$ and $F_k^{\beta} = F\left(P_k^{\beta}, P_{k+1}^{\alpha}\right)$ are computed.

(c) $\Delta P_{k+1}^{\alpha} = \tilde{P}_k^{\alpha} - P_{k+1}^{\alpha}$ is derived; ΔP_{k+1}^{α} has opposite sign to that defined in Eq. S23, yet it does not affect the value of λ_{α} since it is computed as the ratio of s_{α} and c_{α} (Eq. S31, S32 and S34), which linearly depends on ΔP_{k+1}^{α}.

(d) $s_{\beta} = Tr\left[\left[\tilde{F}_{k-1}^{\beta} + J_{k+1}^{\alpha} - J_k^{\alpha}\right]\Delta P_k^{\beta}\right]$ and $c_{\beta} = \frac{1}{2}Tr\left[\left[F_k^{\beta} - \tilde{F}_{k-1}^{\beta} - J_{k+1}^{\alpha} + J_k^{\alpha}\right]\Delta P_k^{\beta}\right]$ are computed.

(e) λ_{β} is set to 1 if $|c_{\beta}| \leq \frac{|s_{\beta}|}{2}$ or $s_{\beta}c_{\beta} \geq 0$; otherwise, λ_{β} is set to $-\frac{s_{\beta}}{2c_{\beta}}$. $\tilde{P}_k^{\beta}$ and $\tilde{F}_k^{\beta}$ are interpolated as

$$\tilde{P}_k^{\beta} = (1 - \lambda_{\beta})\tilde{P}_{k-1}^{\beta} + \lambda_{\beta}P_k^{\beta}$$
$$\tilde{F}_k^{\beta} = (1 - \lambda_{\beta})\tilde{F}_{k-1}^{\beta} + \lambda_{\beta}F_k^{\beta} + (1 - \lambda_{\beta})[J_{k+1}^{\alpha} - J_k^{\alpha}]$$

(f) $\tilde{F}_k^{\beta}$ is diagonalized and P_{k+1}^{β} is calculated within the Aufbau principle.

(g) $J^{\beta}_{k+1} = J\left(P^{\beta}_{k+1}\right)$ and $F^{\alpha}_{k+1} = F\left(P^{\alpha}_{k+1}, P^{\beta}_{k+1}\right)$ are computed.

(h) $\Delta P^{\beta}_{k+1} = \tilde{P}^{\beta}_{k} - P^{\beta}_{k+1}$ is derived.

(i) $E^{UHF}_{k+1}\left(P^{\alpha}_{k+1}, P^{\beta}_{k+1}\right)$ and the eigenvalues as well as the corresponding coefficients of the α- and β-spin orbitals ($\left\{\epsilon^{\alpha}_{\mu,k+1}, \epsilon^{\beta}_{\mu,k+1}\right\}, \left\{C^{\alpha}_{i\mu,k+1}, C^{\beta}_{i\mu,k+1}\right\}$) are computed.

(j) If both of the following two conditions

$$\left|E^{UHF}_{k+1} - E^{UHF}_{k}\right| < 10^{-8} \text{ eV}$$

and

$$max\left\{\left|\epsilon^{\alpha(\beta)}_{\mu,k+1} - \epsilon^{\alpha(\beta)}_{\mu,k}\right|\right\} \leq 10^{-4} \text{ eV}$$

are satisfied, SCF iteration is terminated.

(k) $s_{\alpha} = Tr\left[\left[\tilde{F}^{\alpha}_{k} + J^{\beta}_{k+1} - J^{\beta}_{k}\right]\Delta P^{\alpha}_{k+1}\right]$ and $c_{\alpha} = \frac{1}{2}Tr\left[\left[F^{\alpha}_{k+1} - \tilde{F}^{\alpha}_{k} - J^{\beta}_{k+1} + J^{\beta}_{k}\right]\Delta P^{\alpha}_{k+1}\right]$ are computed.

(l) λ_{α} is set to 1 if $|c_{\alpha}| \leq \frac{|s_{\alpha}|}{2}$ or $s_{\alpha}c_{\alpha} \geq 0$; otherwise, λ_{α} is set to $-\frac{s_{\alpha}}{2c_{\alpha}}$. $\tilde{P}^{\alpha}_{k+1}$ and $\tilde{F}^{\alpha}_{k+1}$ are interpolated as

$$\tilde{P}^{\alpha}_{k+1} = (1-\lambda_{\alpha})\tilde{P}^{\alpha}_{k} + \lambda_{\alpha}P^{\alpha}_{k+1}$$
$$\tilde{F}^{\alpha}_{k+1} = (1-\lambda_{\alpha})\tilde{F}^{\alpha}_{k} + \lambda_{\alpha}F^{\alpha}_{k+1} + (1-\lambda_{\alpha})\left[J^{\beta}_{k+1} - J^{\beta}_{k}\right]$$

(m) k is incremented ($k = k+1$) and the iteration is repeated by going back to step (a).

3. Termination: $C^{\alpha}_{i\mu} = C^{\alpha}_{i\mu,k+1}$, $C^{\beta}_{i\mu} = C^{\beta}_{i\mu,k+1}$, $P^{\alpha} = P^{\alpha}_{k+1}$, $P^{\beta} = P^{\beta}_{k+1}$, $F^{\alpha} = F^{\alpha}_{k+1}$, $F^{\beta} = F^{\beta}_{k+1}$ and $E^{UHF} = E^{UHF}_{k+1}$ are set and stored for following analysis.

S10. Torsional potential profile of PEDOT dimer:

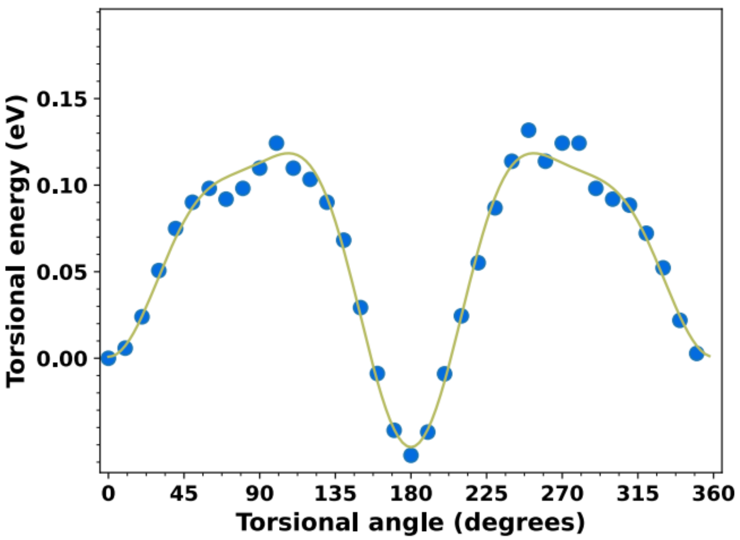

Figure S7: Torsional potential of PEDOT dimer computed from MD simulation outputs of PEDOT-PSS samples (blue filled circle) and fitted within cosine Fourier series with 5 coefficient ($V(\theta) = \frac{A_0}{2} + \sum_{m=1}^{4} A_m \, cos(m\theta)$, θ being the dihedral angle) (green curve). Root mean square error of the fitting is $\sim$6 meV. The analytical expression of the torsional potential is employed to obtain the Boltzmann distribution of θ. In the present study, dihedral angles between PEDOT units in polymer chains are assigned by randomly drawing them from the above obtained Boltzmann distribution.

S11. PEDOT monomer HOMO energy distribution in PEDOT-PSS samples:

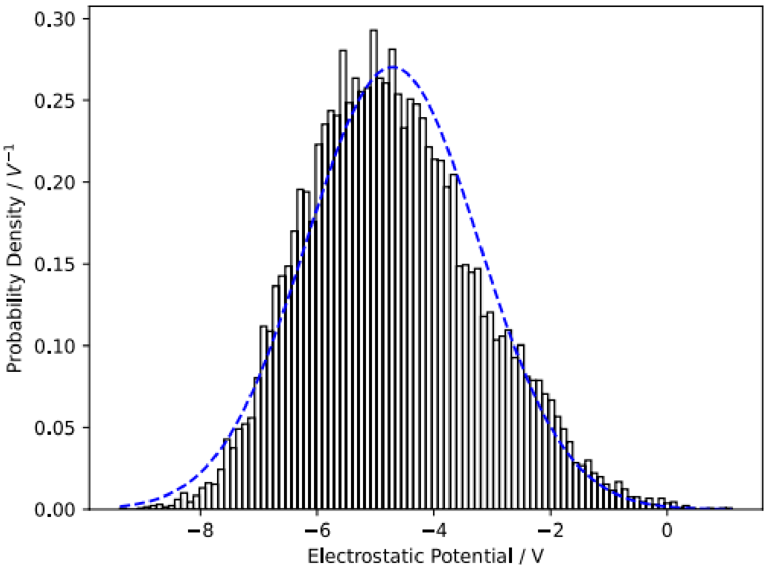

Figure S8: PEDOT monomer HOMO energy distribution (black rectangles) obtained by sampling the electrostatic potential at the center of charge of individual PEDOT moieties in the simulation box. The potential is due to other charged entities in the box but not due to charge-carriers residing on the same PEDOT chain. The distribution can be well-fitted within a Gaussian profile (blue broken line) with the mean at -4.7 V and standard deviation of 1.5 V. Since the HOMO energies contribute to the diagonal elements of the reduced Hamiltonian (Eq. 1 in the main manuscript), a shift in the origin does not affect the final wavefunctions. For arithmetical convenience, we consider a Gaussian distribution with the same standard deviation but centered at zero volts and the HOMO energies of PEDOT monomer units are assigned by randomly drawing them from this distribution.

S12. Distribution of ground state M_S in PEDOT chains of varying length and with varying number of holes:

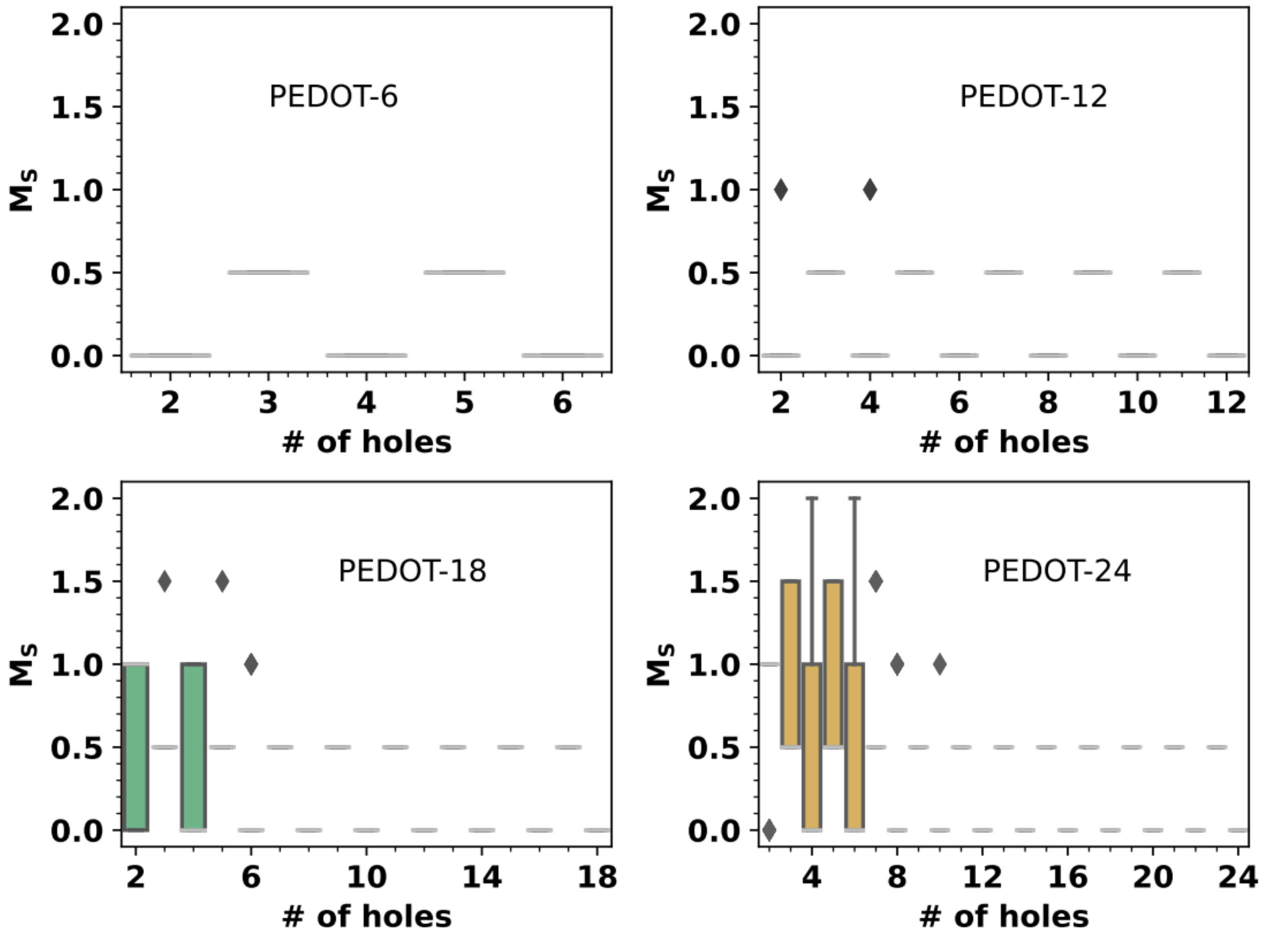

Figure S9: Box plots of ground state M_S distributions of PEDOT chain ensembles of varying length and with varying number of holes. The medians of every distributions are shown by silver colored lines. It is evident from the distributions that the system can frequently assume high spin configurations in longer PEDOT chains with lower number of holes.

S13. Distribution of energy gaps in PEDOT chains of varying length and with varying number of holes:

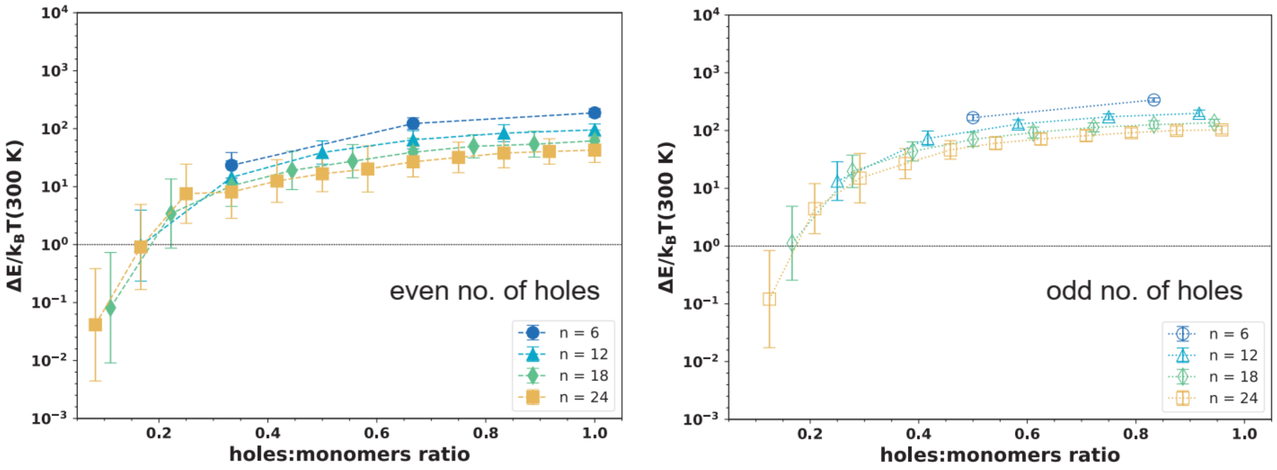

Figure S10: Distribution of energy gaps between ground state spin configuration and next higher energy spin configuration in PEDOT chains of varying length and with varying number of holes; 100 different polymer chain conformations with varying dihedral angles and monomer HOMO energies are considered for a specific chain length and hole density. The left (right) figure shows the energy gaps for even (odd) number of holes. Color and symbol indices are shown in the figure insets. The energy gaps are represented in the units of $k_B T$, $T = 300$ K, and plotted in the logarithmic scale. The black dotted lines represent $\Delta E = k_B T$; other lines connecting the symbols are guides to the eyes.

References:

(1) Coropceanu, V.; Cornil, J.; da Silva Filho, D. A.; Olivier, Y.; Silbey, R.; Brédas, J.-L. Charge Transport in Organic Semiconductors. *Chem. Rev.* **2007**, *107* (4), 926–952. https://doi.org/10.1021/cr050140x.

(2) Prodhan, S.; Manurung, R.; Troisi, A. From Monomer Sequence to Charge Mobility in Semiconductor Polymers via Model Reduction. *Adv. Funct. Mater.* **2023**, *33* (36), 2303234. https://doi.org/10.1002/adfm.202303234.

(3) Pople, J. A.; Beveridge, D. L. *Approximate Molecular Orbital Theory*; McGraw-Hill: New York, 1972.

(4) Szabo, A.; Ostlund, N. S. *Modern Quantum Chemistry: Introduction to Advanced Electronic Structure Theory*, 1. publ., unabr., unaltered republ. of the 1. ed., New York 1989.; Dover Publications: Mineola, New York, 1996.

(5) Kudin, K. N.; Scuseria, G. E. Converging Self-Consistent Field Equations in Quantum Chemistry – Recent Achievements and Remaining Challenges. *ESAIM Math. Model. Numer. Anal.* **2007**, *41* (2), 281–296. https://doi.org/10.1051/m2an:2007022.

(6) Cancès, E.; Le Bris, C. Can We Outperform the DIIS Approach for Electronic Structure Calculations? *Int. J. Quantum Chem.* **2000**, *79* (2), 82–90. https://doi.org/10.1002/1097-461X(2000)79:2<82::AID-QUA3>3.0.CO;2-I.

(7) Kudin, K. N.; Scuseria, G. E.; Cancès, E. A Black-Box Self-Consistent Field Convergence Algorithm: One Step Closer. *J. Chem. Phys.* **2002**, *116* (19), 8255. https://doi.org/10.1063/1.1470195.

(8) Pulay, P. Convergence Acceleration of Iterative Sequences. the Case of Scf Iteration. *Chem. Phys. Lett.* **1980**, *73* (2), 393–398. https://doi.org/10.1016/0009-2614(80)80396-4.

(9) Pulay, P. Improved SCF Convergence Acceleration. *J. Comput. Chem.* **1982**, *3* (4), 556–560. https://doi.org/10.1002/jcc.540030413.

(10) Garza, A. J.; Scuseria, G. E. Comparison of Self-Consistent Field Convergence Acceleration Techniques. *J. Chem. Phys.* **2012**, *137* (5), 054110. https://doi.org/10.1063/1.4740249.

(11) Cancès, E.; Le Bris, C. On the Convergence of SCF Algorithms for the Hartree-Fock Equations. *ESAIM Math. Model. Numer. Anal.* **2000**, *34* (4), 749–774. https://doi.org/10.1051/m2an:2000102.

(12) Yamamoto, J.; Mochizuki, Y. Optimal Damping Algorithm for Unrestricted Hartree-Fock Calculations. *Chem-Bio Inform. J.* **2014**, *14* (0), 14–33. https://doi.org/10.1273/cbij.14.14.
